# Association between laryngoplasty and pneumonia incidence in patients with unilateral vocal fold paralysis: A Japanese insurance claims database study

**DOI:** 10.1371/journal.pone.0352874

**Published:** 2026-07-02

**Authors:** Masahiro Hemmi, Daisuke Sano, Reo Tanoshima, Nobuhiko Oridate

**Affiliations:** 1 Department of Otorhinolaryngology-Head and Neck Surgery, University, School of Medicine, Kanazawa-ku, Yokohama, Kanagawa, Japan; 2 Department of Health Data Science, Yokohama City University, Graduate School of Data Science, Nishi-ku, Yokohama, Kanagawa, Japan; Public Library of Science, UNITED KINGDOM OF GREAT BRITAIN AND NORTHERN IRELAND

## Abstract

**Objective:**

Unilateral vocal fold paralysis (UVFP) can lead to both voice impairment and dysphagia. Previous studies have suggested that surgeries aimed at improving voice function in patients with UVFP may also incidentally enhance swallowing. However, the preventive effect of such surgeries on pneumonia remains unclear. We evaluated the impact of laryngoplasty on the incidence of pneumonia in Japan using a large employment insurance claims database of corporate employees under 75 years of age and their family members.

**Methods:**

The study cohort consisted of patients diagnosed with UVFP between January 2013 and December 2022, identified from an insurance claims database. A propensity score–matched cohort was created using a 1:3 matching ratio between the treatment and non-treatment groups. Follow-up began at the index date, defined as the initial diagnosis of UVFP, and continued until the end of the study period. The primary analysis compared the cumulative incidence of pneumonia between treatment and non-treatment groups. The secondary analysis employed a self-controlled design to compare pneumonia incidence before and after laryngoplasty within treated patients.

**Results:**

The full cohort included 7,641 patients with UVFP, of whom 914 comprised the matched cohort (treatment group, n = 230; non-treatment group, n = 684). The cumulative incidence of pneumonia tended to be higher in the treatment group than in the non-treatment group (hazard ratio, 1.39; 95% CI, 0.93–2.08; p = 0.098). In contrast, the self-controlled analysis demonstrated a lower pneumonia incidence after laryngoplasty compared with the pre-treatment period (incidence rate ratio, 0.36; 95% CI, 0.27–0.52; p < 0.001).

**Conclusions:**

Given the discordant findings across analytical approaches, no definitive conclusion regarding a preventive effect can be drawn from the present study. Further studies are needed to evaluate long-term outcomes and to clarify causal relationships.

## Introduction

Unilateral vocal fold paralysis (UVFP), characterized by fixation of the affected vocal fold in a paramedian or lateral position, can result in glottic insufficiency and voice impairment. UVFP occurs on the left side in approximately 80%–95% of cases [1–3]. The etiology of UVFP includes iatrogenic injury, tumors, idiopathic causes, neurological disorders, and trauma [4,5], with iatrogenic injury being the most common [6]. In particular, recurrent laryngeal nerve paralysis frequently arises as a complication of tracheal intubation, thyroid surgery, or cardiothoracic procedures [5]. In addition to voice impairment, UVFP can cause swallowing dysfunction due to impaired laryngeal elevation and reduced pharyngeal pressure [7]. It may also weaken the cough reflex and compromise airway protection, thereby increasing the risk of aspiration pneumonia [8,9]. In fact, dysphagia has been reported in 55%–69% of patients with UVFP [10,11], and aspiration is observed in 20%–50% of cases [11,12].

Initial management typically involves conservative treatment with voice therapy for 6–12 months to allow for potential spontaneous recovery or compensatory movement of the contralateral vocal fold [13,14]. If functional recovery is inadequate, surgical intervention, collectively referred to as laryngoplasty, is considered to restore voice quality [15]. Laryngoplasty includes injection laryngoplasty (IL) and laryngeal framework surgery (LFS) [16,17], with the choice of procedure guided by the overall health status of the patient and the severity of the paralysis. A meta-analysis by Coulter et al. reported improvement in both subjective and objective measures of swallowing dysfunction in 90% of patients undergoing IL and 92% undergoing LFS [12]. However, evidence regarding the impact of laryngoplasty on the incidence of pneumonia remains limited [18,19].

Understanding the clinical outcomes of laryngoplasty may help determine the indications for surgical treatment in the management of UVFP. In this study, we conducted a cross-institutional analysis using a Japanese insurance claims database to evaluate the association between laryngoplasty and pneumonia incidence in patients with UVFP.

## Materials and methods

### Data source

In this retrospective cohort study, we utilized a large employment insurance claims database maintained by JMDC Inc. The JMDC database comprises cumulative patient records from approximately 17 million individuals, primarily company employees aged <75 years and their dependents [20,21]. Operational since 2009, the database includes demographic characteristics (e.g., age, sex), pharmacy claims, disease diagnoses coded using the International Classification of Diseases, 10th Revision (ICD-10), medical procedures, and hospitalization records [22]. A representative cohort of patients with UVFP was identified using this database, and treatment histories were tracked longitudinally across multiple medical institutions. To protect patient privacy, all personally identifiable information was anonymized and encrypted.

### Study design, setting, and outcomes

**Fig 1:**
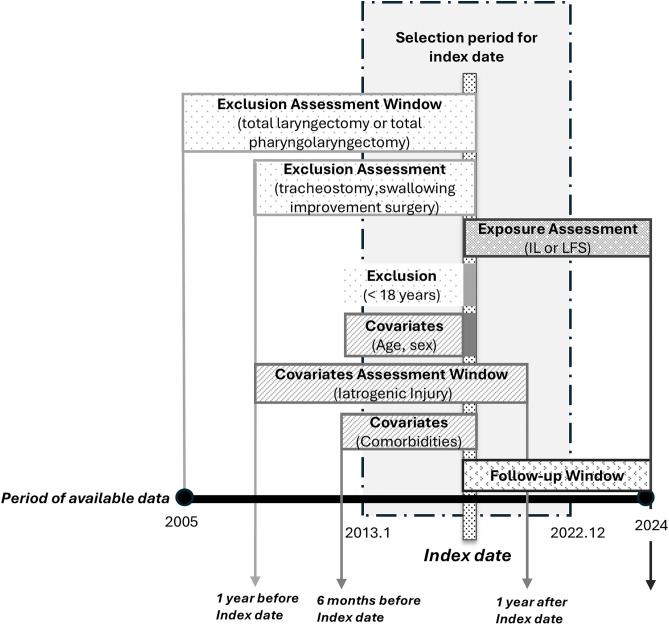
Study design. This figure illustrates the timeline for exclusion criteria, covariates, exposure, and follow-up assessment windows used in the study. The black solid line indicates the period of available data. The study selection period was from January 1, 2013, to December 31, 2022. The index date was defined as the first recorded diagnosis of unilateral vocal fold paralysis within the selection period. Exclusion criteria assessed prior to the index date included history of total laryngectomy or total pharyngolaryngectomy, tracheostomy, swallowing improvement surgery, and age under 18 years. Covariates such as iatrogenic injury and comorbidities were evaluated within specified pre-index periods. The exposure window for injection laryngoplasty (IL) or laryngeal framework surgery (LFS) was defined as occurring after the index date. The follow-up period extended from the index date to the end of the available data period.

This study was conducted in accordance with the Strengthening the Reporting of Observational Studies in Epidemiology (STROBE) guidelines [23]. The data extraction period extended from January 2005 to January 2024. The study selection period was defined as January 1, 2013, to December 31, 2022. The index date was the first recorded diagnosis of UVFP (ICD-10 code J380) within the selection period. Demographic variable　were assessed at the index date, while comorbidities were collected during a 6-month baseline period preceding the index date. To identify iatrogenic causes of UVFP, surgical histories potentially associated with vocal fold paralysis—including thyroid, cardiac, esophageal, and lung surgeries—were extracted from medical claims codes recorded within 1 year before and after the index date. The medical billing codes for laryngoplasty included injection laryngoplasty (IL) [150106410] and laryngeal framework surgery (LFS) [150109110, 150109310, 150398710]. Patients who underwent IL or LFS at least once during the selection period were categorized into the treatment group, whereas those who did not undergo either procedure formed the non-treatment group. Patients younger than 18 years, those with a baseline covariate assessment period of less than six months, and those who underwent tracheostomy [150106210], surgical procedures for dysphagia improvement [150345110, 150345210, 150345310, 150345310, 150345310], total laryngectomy [150345410, 150107910], or total pharyngolaryngectomy [150108210] were excluded from the study. The follow-up period began at the index date and continued until death, withdrawal from the employee’s health insurance, or the end of the data extraction period. An overview of the study design is provided in the schematic diagram (Fig 1).

As the primary analysis, we compared the cumulative incidence of pneumonia between the treatment group and the non-treatment group, with follow-up starting from a predefined index date, defined as the onset of UVFP. This analysis aimed to assess potential differences in pneumonia risk at the population level between patients who underwent laryngoplasty and those who did not. As the secondary analysis, we conducted a self-controlled analysis within the treatment group to compare the incidence of pneumonia before and after laryngoplasty. This analysis aimed to evaluate within-person changes in pneumonia incidence. For patients who underwent multiple IL or LFS procedures, the latest available surgery date was used as the reference point. The pre-treatment period was defined as the time from the index date to the reference point, and the post-treatment period as the time from the reference point to the end of follow-up. Pneumonia incidence rates were compared between these two periods. Subgroup analyses of the secondary outcome were conducted by surgical procedure (IL vs. LFS). In addition, as an exploratory analysis, we examined baseline predictors of pneumonia in the overall cohort. This analysis aimed to identify vulnerable subpopulations at higher risk of pneumonia among patients with UVFP and to inform appropriate patient selection for future preventive interventions.

Pneumonia was defined based on the following criteria: (1) ICD-10 diagnosis codes J12–J18 and J69, and (2) concurrent prescription of antibiotics. (supplementary material, S1 Table). Each pneumonia episode accompanied by antibiotic prescription was considered an independent recurrent event during the follow-up period, and the number of such events was analyzed as count data in the primary analysis. This algorithm in the JMDC database has been used in previous studies [21,24].

### Statistical analysis

Descriptive statistics were used to summarize patient characteristics. Continuous variables are presented as means with standard deviations (SD), and categorical variables as frequencies and proportions. Propensity score (PS) matching at a 1:3 ratio was performed using a caliper width of 0.2 on the logit scale of the standard deviation to adjust for baseline differences between the treatment and non-treatment groups [25]. The matching of variables was considered balanced when the standardized mean differences (SMDs) between groups were less than 0.1 [26]. The balance was also visually assessed using a Love plot. The PS was calculated using a multivariable logistic regression model that integrated Age, Sex, Comorbidities (Asthma, Chronic obstructive pulmonary disease, Diabetes mellitus, Heart failure, Stroke, Cancer, Dysphagia), and Cause of UVFP. Comorbidities were also identified according to ICD-10 codes listed in the supplementary material (S1 Table). PS matching was performed using the *MatchIt* package in R. The cumulative incidence of pneumonia post-index date was estimated using the Kaplan–Meier method in the population after matching, with between-group comparisons performed using the log-rank test. The incidence rate (IR) and incidence rate ratios (IRRs) with 95% confidence intervals (CIs) of pneumonia were calculated in the treatment group based on the number of events per person-year. The IRRs were estimated using Poisson regression models.

If any covariates remained insufficiently balanced after PS matching, we performed additional adjustment using multivariable Cox proportional hazards models including these covariates, as recommended in previous studies to mitigate residual confounding in matched observational studies [27,28]. The self-controlled comparison of pneumonia incidence before and after laryngoplasty may be susceptible to confounding if pneumonia events occurring shortly before surgery influence the indication for laryngoplasty. Therefore, as a sensitivity analysis, we excluded pneumonia events occurring within the 2 months immediately preceding laryngoplasty, along with the corresponding person-time. In addition, to account for potential immortal time bias related to differential censoring or loss to follow-up, we performed a separate sensitivity analysis by excluding cases in which the post-treatment observation period was shorter than the pre-treatment period. Data extraction was performed using SQLite version 3.13.1, and statistical analyses were conducted using R version 4.4.1 (R Foundation for Statistical Computing, Vienna, Austria). A two-sided *p*-value < 0.05 was considered statistically significant.

### Ethics considerations

All procedures involving human participants were conducted in accordance with the 1964 Declaration of Helsinki and its later amendments or comparable ethical standards. This study was approved by the Institutional Review Board of Yokohama City University (approval number: F250100033). The requirement for obtaining informed consent was waived by the ethics committee because this retrospective observational study used anonymized secondary data. The data were accessed for research purposes on February 5, 2025, after receiving approval from the institutional review board. The authors did not have access to information that could identify individual participants during or after data collection.

## Results

### Cohort and patients characteristics

**Fig 2:**
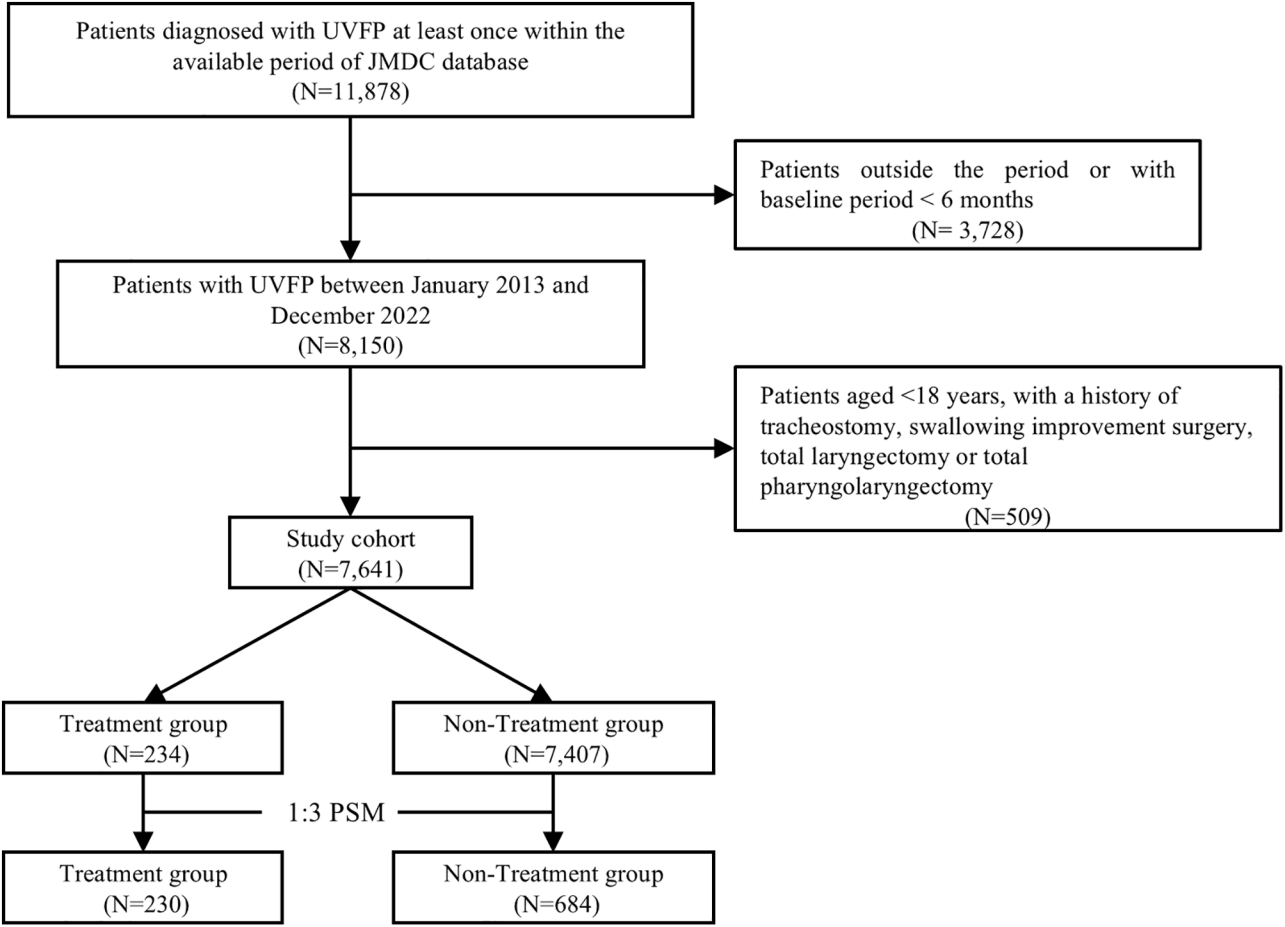
Flowchart of patient selection. Patients with UVFP were extracted from the JMDC database. After applying the inclusion and exclusion criteria, 7,641 patients were eligible for the study and were stratified into treatment and non-treatment groups based on whether they received IL and/or LFS. Following 1:3 PS matching, 230 patients were assigned to the treatment group and 684 to the non-treatment group. Abbreviations: UVFP, unilateral vocal fold paralysis; JMDC, Japan Medical Data Center; IL, injection laryngoplasty; LFS, laryngeal framework surgery.

During the JMDC claims database utilization period, 11,878 patients were identified with a diagnosis of UVFP. Among these, 8,150 patients whose diagnosis date fell within the predefined selection period were screened for eligibility (Fig 2). After applying the eligibility criteria, 7,641 patients were included in the initial cohort. Following 1:3 PS matching, 230 patients were assigned to the treatment group and 684 to the non-treatment group.

The demographic and clinical characteristics of the study cohort before and after PS matching are summarized (Table 1). There was no significant difference in age distribution between the two groups. In the unmatched cohort, the treatment group had a higher proportion of male patients, as well as a higher prevalence of lung cancer, pneumonia and dysphagia than the non-treatment group. After PS matching, SMDs were generally reduced, indicating improved covariate balance between groups, although some variables, such as stroke and dysphagia, still exceeded the 0.1 threshold (supplementary material, S1 Fig). The PS model had an area under the curve (AUC) of 0.667, demonstrating moderate discriminatory ability. As shown in Table 1, the etiology of UVFP was unknown in most cases. Iatrogenic injury accounted for 19.7% of cases in the treatment group and 26.1% in the non-treatment group before matching, and 18.3% and 17.3% after matching, respectively. Among iatrogenic cases, thyroid surgery was the predominant cause, representing 9.0% in the treatment group and 21.5% in the non-treatment group before matching, and 9.1% and 9.8% after matching, respectively.

**Table 1:** Patient baseline characteristics before and after PS matching.

	Before PS matching			After PS matching		
Characteristic	Treatment group N = 234	Non-treatment group N = 7,407	SMD	Treatment group N = 230	Non-treatment group N = 684	SMD
**Age (SD)**	49.35 (13.21)	49.43 (12.82)	0.006	49.20 (13.25)	48.68 (13.17)	0.039
**Age category**						
18–50 yr (%)	117 (50.0)	3636 (49.1)	0.018	116 (50.4)	350 (51.2)	0.015
≧ 51 yr (%)	117 (50.0)	3771 (50.9)		114 (49.6)	334 (48.8)	
**Sex**						
Male (%)	134 (57.3)	3354 (45.3)	0.241	132 (57.4)	391 (57.2)	0.005
Female (%)	100 (42.7)	4053 (54.7)		98 (42.6)	293 (42.8)	
**Comorbidities**						
Asthma (%)	33 (14.1)	774 (10.4)	0.111	33 (14.3)	105 (15.4)	0.028
CKD (%)	3 (1.3)	103 (1.4)	0.009	3 (1.3)	5 (0.7)	0.057
COPD (%)	7 (3.0)	184 (2.5)	0.031	7 (3.0)	22 (3.2)	0.01
Diabetes mellitus (%)	79 (33.8)	2187 (29.5)	0.091	77 (33.5)	220 (32.2)	0.028
Heart failure (%)	31 (13.2)	1301 (17.6)	0.12	30 (13.0)	75 (11.0)	0.064
Stroke (%)	14 (6.0)	420 (5.7)	0.013	14 (6.1)	19 (2.8)	0.161
Cancer						
Head and neck cancer (%)	62 (26.5)	2111 (28.5)	0.045	61 (26.5)	169 (24.7)	0.042
Lung cancer (%)	36 (15.4)	582 (7.9)	0.237	32 (13.9)	85 (12.4)	0.044
Thyroid cancer (%)	31 (13.2)	1571 (21.2)	0.212	30 (13.0)	90 (13.2)	0.003
Esophageal cancer (%)	8 (3.4)	272 (3.7)	0.014	8 (3.5)	13 (1.9)	0.098
Pneumonia (%)	29 (12.4)	668 (9.0)	0.109	29 (12.6)	84 (12.3)	0.01
Dysphagia (%)	29 (12.4)	411 (5.5)	0.241	28 (12.2)	62 (9.1)	0.101
**Cause of UVFP**						
Iatrogenic injury (%)	46 (19.7)	1936 (26.1)	0.155	42 (18.3)	118 (17.3)	0.026
Thyroid surgery (%)	21 (9.0)	1591 (21.5)	0.353	21 (9.1)	67 (9.8)	0.023
Cardiac surgery (%)	10 (4.3)	212 (2.9)	0.076	10 (4.3)	23 (3.4)	0.051
Esophagus surgery (%)	9 (3.8)	62 (0.8)	0.2	5 (2.2)	11 (1.6)	0.042
Lung surgery (%)	7 (3.0)	101 (1.4)	0.112	6 (2.6)	19 (2.8)	0.01
Unknown (%)	188 (80.3)	5471 (73.9)	0.155	188 (81.7)	566 (82.7)	0.026

Abbreviations: PS, propensity score; SMD, standardized mean difference; SD, standard deviation; CKD, chronic kidney disease; COPD, chronic obstructive pulmonary disease; UVFP, unilateral vocal fold paralysis

### Details of laryngoplasty procedures

Details of the laryngoplasty procedures in the matched treatment group are presented (Table 2). Among the 230 patients in the treatment group, 104 underwent injection IL only, 104 underwent LFS only, and 22 received both IL and LFS. The median number of procedures was one for patients undergoing either IL (range: 1–7) or LFS (range: 1–5), and three for those receiving both treatments (range: 2–6). The median pre-treatment period was 108 days for IL (range: 0–1,429 days), 178 days for LFS (range: 0–2,519 days), and 455 days for combined IL and LFS (range: 33–1,541 days). The median post-treatment period was 561 days for IL (range: 4–3,460 days), 731 days for LFS (range: 72–3,121 days), and 293 days for combined IL and LFS (range: 49–1,350 days).

**Table 2:** Details of laryngoplasty procedures.

	IL	LFS	IL + LFS
Number of patients	104	104	22
Number of procedures, median (range)	1 (1-7)	1 (1-5)	3(2-6)
*Pre-treatment period, median (range)	108 (0-1,429)	178 (0-2,519)	455 (33−1,541)
**Post-treatment period, median (range)	561 (4−3,460)	731 (72−3,121)	293 (49−1,350)

* The pre-treatment period was defined as the number of days from the index date to the reference point.

**The post-treatment period was the number of days from the reference point to the end of follow-up.

Abbreviations: IL, injection laryngoplasty; LFS, laryngeal framework surgery

### Pneumonia incidence

**Fig 3:**
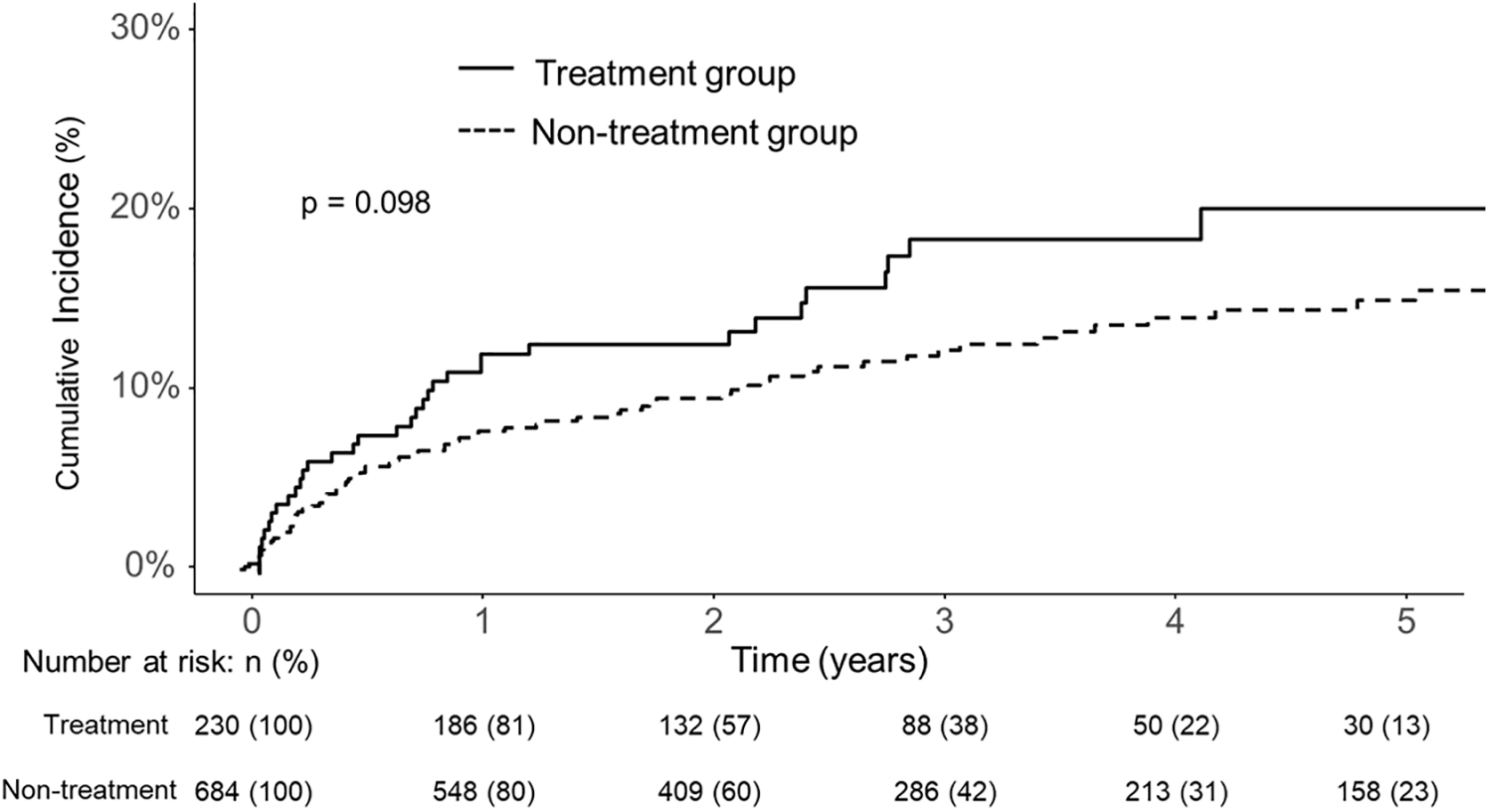
Cumulative incidence of pneumonia in the treatment and non-treatment groups. The treatment group exhibited a higher cumulative incidence of pneumonia over time following the index date compared to the non-treatment group after PS matching, although this difference did not reach statistical significance (hazard ratio [HR], 1.39; 95% CI, 0.93–2.08; p = 0.098). The figures below indicate the number of patients at risk at each time point. Abbreviations: PS, propensity score; CI, confidence interval.

The cumulative incidence of pneumonia following the index date in the matched cohort is shown (Fig 3). The median observation period was 2.17 years in the treatment group and 2.44 years in the non-treatment group. At five years, the cumulative incidence of pneumonia was higher in the treatment group (18.6%) than in the non-treatment group (14.4%), although this difference did not reach statistical significance (hazard ratio [HR], 1.39; 95% CI, 0.93–2.08; *p* = 0.098). These findings suggest a trend toward a higher risk of pneumonia in the treatment group compared with the non-treatment group over the entire study period.

**Fig 4:**
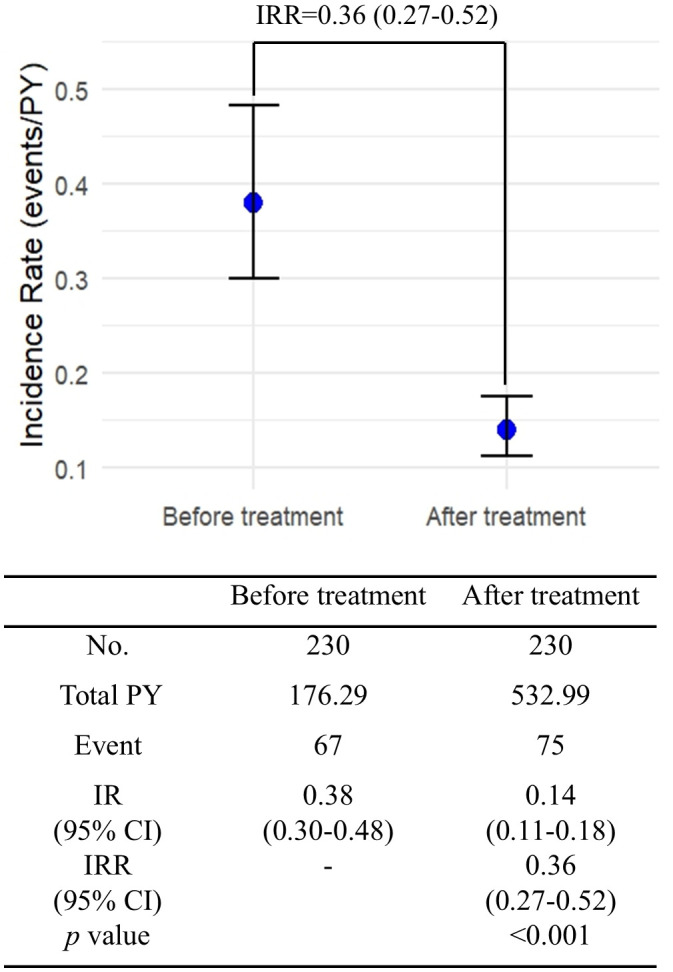
Incidence rate of pneumonia before and after surgery. The IRR after surgery was significantly lower than that before surgery in the treatment group (IRR,0.36; 95% CI: 0.27–0.52; p < 0.001). Abbreviations: IR, incidence rate; PY, person-year; IRR, incidence rate ratio; CI, confidence interval..

The IRR before and after surgery were evaluated in the treatment group (Fig 4). The IRR after surgery was significantly lower than that before surgery (0.36; 95% CI: 0.27–0.52; *p* < 0.001). In the subgroup analysis by surgical procedure, both IL (n = 104) and LFS (n = 104) were associated with a reduction in pneumonia incidence after treatment (S2 Table). The IRR decreased from 0.34 to 0.16 for IL (IRR, 0.47; 95% CI, 0.28–0.79; *p* = 0.003) and from 0.22 to 0.12 for LFS (IRR, 0.54; 95% CI, 0.31–0.97; *p* = 0.031). There was no significant difference between IL and LFS in postoperative cumulative incidence (S2 Fig). The IRRs of pneumonia risk factors in the matched cohort were estimated by the Poisson regression model (S3 Table). Age and dysphagia were identified as significant risk factors for pneumonia, with dysphagia showing a particularly strong association (IRR 4.12; 95% CI, 3.41–4.96) and older age also being associated with increased risk (IRR 2.87; 95% CI, 2.31–3.35).

### Sensitivity analysis

In multivariable Cox proportional hazards models that additionally adjusted for stroke and dysphagia, which remained imbalanced after PS matching (SMD > 0.1), the treatment group tended to have a higher incidence of pneumonia compared with the non-treatment group; however, this difference was not statistically significant (adjusted HR, 1.35; 95% CI, 0.90–2.01; p = 0.149) (S4 Table). Of the 230 patients in the treatment group, 67 were excluded from the analysis because their preoperative observation period was shorter than 2 months. Among the remaining 163 patients, comparison of the period from the index date to 2 months before surgery with the postoperative observation period showed that the incidence of pneumonia was significantly lower after laryngoplasty than before surgery (IRR 0.55; 95% CI, 0.37–0.83; p = 0.004) (S3 Fig). In a separate sensitivity analysis addressing potential immortal time bias, 53 patients were excluded due to a post-treatment period shorter than the pre-treatment period. Among the remaining 177 patients, the IRR of pneumonia after surgery was significantly lower than that before surgery (IRR 0.25; 95% CI: 0.17–0.37; p < 0.001) (S4 Fig).

## Discussion

This retrospective observational study is the first to investigate the clinical characteristics of patients with UVFP aged 18–75 years and to examine the impact of laryngoplasty on pneumonia incidence using a large insurance claims database in Japan. In the matched cohort analysis, the incidence of pneumonia after UVFP onset tended to be higher in the treatment group than in the non-treatment group (HR, 1.39; 95% CI, 0.93–2.08; p = 0.098). In contrast, the self-controlled analysis demonstrated a reduction in pneumonia incidence after laryngoplasty compared with the preoperative period within the same individuals (IRR, 0.36; 95% CI: 0.27–0.52; p < 0.001). These findings indicate that the estimated association between laryngoplasty and pneumonia risk is sensitive to the analytical framework applied, and a definitive conclusion regarding risk reduction cannot be drawn from the present data.

A prior study utilizing Taiwan’s National Health Insurance Database reported a lower incidence of pneumonia in the laryngoplasty group with UVFP than in the untreated group (HR, 0.49; 95% CI, 0.27–0.90; *p* = 0.021) [19]. The discrepancy may result from differences in patient populations. Particularly, the present study included patients with more severe cases who were at higher risk of developing pneumonia reflecting real-world clinical practice, as several Japanese studies have reported the therapeutic efficacy of laryngoplasty in UVFP patients with an aspiration risk [29–31]. In addition, variations in the definition of pneumonia and in the classification of surgical procedures may have led to differences in event ascertainment. Barnes et al. investigated the effects of early injection laryngoplasty on pneumonia prevention within six months after cardiothoracic surgery in patients with UVFP. Their findings showed a significantly lower incidence of pneumonia in patients who received IL compared to those who did not (HR, 0.33; 95% CI, 0.11–0.98; *p* = 0.045) [18]. Nevertheless, the short follow-up period and lack of a pre–post comparison design limited the evaluation of long-term treatment effects. Thus, our study adds to the existing literature by examining the relationship between laryngoplasty and pneumonia incidence using multiple analytical approaches within a large insurance database.

In the primary matched cohort analysis, stroke and dysphagia—both clinically important factors that may influence pneumonia risk—remained imbalanced after PS matching (SMD > 0.1). Therefore, we performed additional adjustment using multivariable Cox proportional hazards models including these covariates. Although the adjusted analysis yielded results consistent in direction with those of the matched cohort analysis, residual imbalance in measured covariates was unlikely to materially alter the between-group association. Regarding the self-controlled analysis, pneumonia episodes occurring shortly before laryngoplasty may influence the decision to perform surgery, resulting in event-dependent exposure and a potential overestimation of preoperative pneumonia incidence. A sensitivity analysis excluding pneumonia events within the 2 months immediately preceding surgery attenuated the magnitude of the reduction (IRR increased from 0.36 to 0.55), suggesting that the observed effect may partially reflect perioperative time-related biases. In addition, several strong time-varying factors—such as progression of dysphagia, increasing frailty, and changes in clinical surveillance—are likely to differ systematically between the pre- and post-surgical periods. Because these factors were not available in the present database, the observed reduction in pneumonia incidence before and after laryngoplasty should be interpreted with caution.

In the Poisson regression analysis, age (≥51 years) and dysphagia were identified as significant risk factors for pneumonia. In particular, dysphagia events during baseline period showed the strongest association (IRR, 4.12; 95% CI, 3.41–4.96). Cancer was also relatively associated with a potential increase in pneumonia risk (IRR, 1.12; 95% CI, 0.94–1.32), as previous studies have also reported a higher incidence of pneumonia in patients with head and neck cancer, esophageal cancer, and lung cancer [32]. Older age is consistently associated with an increased risk of pneumonia, partly due to age-related declines in immune function, decreased cough reflex sensitivity, and reduced mucociliary clearance [33,34]. Dysphagia is a well-established predictor of pneumonia, as impaired swallowing mechanisms can lead to silent aspiration and recurrent lower respiratory tract infections [35]. Patients with UVFP may exhibit supraglottic and pharyngeal abnormalities, such as reduced laryngeal elevation and pharyngeal residue, which can lead to aspiration [36,37]. Recent study demonstrated how sudden relief of upper airway obstruction can precipitate acute pulmonary compromise due to abrupt shifts in intrathoracic and vascular pressures. These findings underscore the critical importance of maintaining airway stability to prevent secondary respiratory complications [38]. In line with this pathophysiological understanding, controlled and sustained restoration of airway function plays a preventive role in mitigating pulmonary morbidity and the observed within-person reduction in pneumonia incidence after laryngoplasty may be consistent with a potential protective effect.

Laryngoplasty restores glottic function by minimizing the glottic gap, thereby reducing the risk of aspiration [37]. Additionally, it enhances subglottic pressure, promoting effective sputum clearance [39]. Watanabe et al. reported that the combination of arytenoid adduction and medialization laryngoplasty significantly improved cough peak flow in patients with UVFP [40]. This finding suggests that laryngoplasty not only strengthens cough through increased intrathoracic pressure but also helps restore airway protection [41]. Moreover, Bruno et al. demonstrated that in UVFP patients with dysphagia, residual pressure at the upper esophageal sphincter measured by high-resolution manometry increased after laryngoplasty [42]. This finding indicates that improvement in subglottic pressure may enhance intrabolus pressure generation, playing a critical role in restoring pharyngeal clearance and reducing the risk of aspiration-related pneumonia [31]. Although laryngoplasty is beneficial for patients with recurrent laryngeal nerve paralysis, structural modifications cannot restore laryngeal sensation, which is essential for the sequence of swallowing; therefore, improvement in swallowing function is often limited [31]. Adjunctive interventions such as swallowing therapy and pulmonary rehabilitation may influence pneumonia risk in patients with UVFP [43,44]. Because information on rehabilitation and underlying causes of non-iatrogenic paralysis was not available in the present database, we could not account for these factors. Therefore, postoperative management may have partly contributed to the observed within-person reduction in pneumonia incidence.

IL is a minimally invasive outpatient procedure that achieves temporary medialization of the vocal folds, commonly using injectable materials such as hyaluronic acid or autologous fat [16,39]. In contrast, LFS, which includes type I thyroplasty and/or arytenoid adduction, is a more invasive procedure intended to achieve permanent vocal fold adduction using materials such as Gore-Tex or titanium implants [45,46]. Owing to its procedural simplicity, IL is often performed in outpatient clinical settings and is sometimes used as an initial treatment before considering LFS [47,48]. However, the effect of injected hyaluronic acid filler is generally reported to last for only 4–6 months, after which natural absorption may lead to recurrence of aspiration [49]. Therefore, it is desirable to conduct objective evaluations such as fiberoptic endoscopic evaluation of swallowing (FEES) or video fluoroscopic swallow study (VFSS) before and after surgery to assess swallowing function and the presence of aspiration over time.[50]A meta-analysis of 275 patients with UVFP reported that those who underwent IL within six months of onset were significantly less likely to require subsequent LFS compared to patients receiving conservative treatment (relative risk = 0.25; 95% CI, 0.14–0.45) [51]. In the present study, IL and LFS procedures were identified based on medical claims codes, and the median interval from onset to laryngoplasty was 108 days for IL and 178 days for LFS. In the subgroup analysis by surgical procedure, both IL and LFS demonstrated lower IRRs after treatment—0.47 (95% CI, 0.28–0.79) for IL and 0.54 (95% CI, 0.31–0.97) for LFS. These results suggest that performing IL or LFS within approximately six months of onset may be associated with a lower subsequent pneumonia incidence among high-risk individuals with UVFP.

This study has several limitations. First, the use of insurance claims data does not ensure complete diagnostic accuracy for either UVFP or pneumonia. In some cases, the pre-treatment observation period was zero, suggesting that the onset of UVFP may have preceded the recorded index date. This limitation may have complicated causal inference regarding the association between laryngoplasty and reductions in pneumonia incidence. With respect to the outcome definition, pneumonia was identified using an algorithm based on diagnostic codes combined with systemic antibiotic prescriptions; however, this algorithm has not been fully validated in the JMDC database, and the possibility of outcome misclassification cannot be excluded. Second, detailed clinical information, such as the severity of UVFP or pneumonia, was not available in the claims database. These factors are likely to strongly influence the likelihood of undergoing laryngoplasty, and the presence of unmeasured confounding may have resulted in a preferential selection of more severe patients into the treatment group. Consequently, the observed reduction in pneumonia incidence may, in part, reflect regression to the mean rather than a pure treatment effect. Third, self-controlled analysis may be susceptible to time-varying confounding and event-dependent exposure. Pneumonia episodes occurring shortly before surgery may have influenced the indication for laryngoplasty and patients’ clinical status may have differed systematically between the pre- and post-surgical periods. These limitations may have biased within-person comparisons. As described previously, the observed reduction in pneumonia incidence after surgery should therefore be interpreted with caution. Fourth, individuals aged >75 years were not included in the database, potentially excluding a population with a higher prevalence of cancer-related or iatrogenic UVFP, which may have influenced our results. Finally, as the study population was limited to patients in Japan, the generalizability of these findings to other populations may be limited. Despite these limitations, this study provides large-scale real-world evidence regarding the association between laryngoplasty and pneumonia incidence in patients with UVFP. Notably, the direction of the association differed depending on the analytical framework applied, with discordant findings observed between the matched cohort and self-controlled analyses. Therefore, no definitive conclusion regarding the preventive effect of laryngoplasty on pneumonia can be drawn from the present study. Nevertheless, the use of a large and representative sample provides valuable real-world insights into treatment patterns and pneumonia risk among patients with UVFP. Further studies employing more rigorous causal designs are needed to clarify the true clinical impact of laryngoplasty.

## Conclusions

This retrospective cohort study, based on a large Japanese insurance claims database, examined the association between laryngoplasty and pneumonia incidence in patients younger than 75 years with UVFP. In the matched cohort analysis, pneumonia incidence tended to be higher in the treatment group, whereas the self-controlled analysis demonstrated a reduction in pneumonia incidence after laryngoplasty within the same individuals. These findings indicate that the observed association depends on the analytical approach applied. Further studies are needed to clarify long-term outcomes and causal relationships.

## Supporting information

S1 Fig Love plot showing standardized mean differences before and after propensity score matching. (PDF)

S2 Fig Postoperative cumulative incidence of pneumonia by surgical procedure (IL and LFS). (PDF)

S3 Fig Incidence rate of pneumonia before and after surgery in the sensitivity analysis. (PDF)

S4 Fig Incidence rate of pneumonia before and after surgery in the sensitivity analysis. (PDF)

S1 Table ICD-10 codes and WHO-ATC codes for disease and drug definition. (PDF)

S2 Table Incidence rate of pneumonia before and after surgery in the subgroup analysis by surgical procedure (IL and LFS). (PDF)

S3 Table Incidence rate ratio estimation of pneumonia risk factors by poisson regression model. (PDF)

S4 Table Pneumonia incidence estimated using Cox proportional hazards models in the propensity score–matched cohort. (PDF)
