# Supplementary material for: Association between laryngoplasty and pneumonia incidence in patients with unilateral vocal fold paralysis: A Japanese insurance claims database study
Source: PLoS One. 2026 Jul 2;21(7):e0352874. doi: 10.1371/journal.pone.0352874 (PMC13327127; doi:10.1371/journal.pone.0352874)

S1 Fig. Love plot showing standardized mean differences before and after propensity score matching.

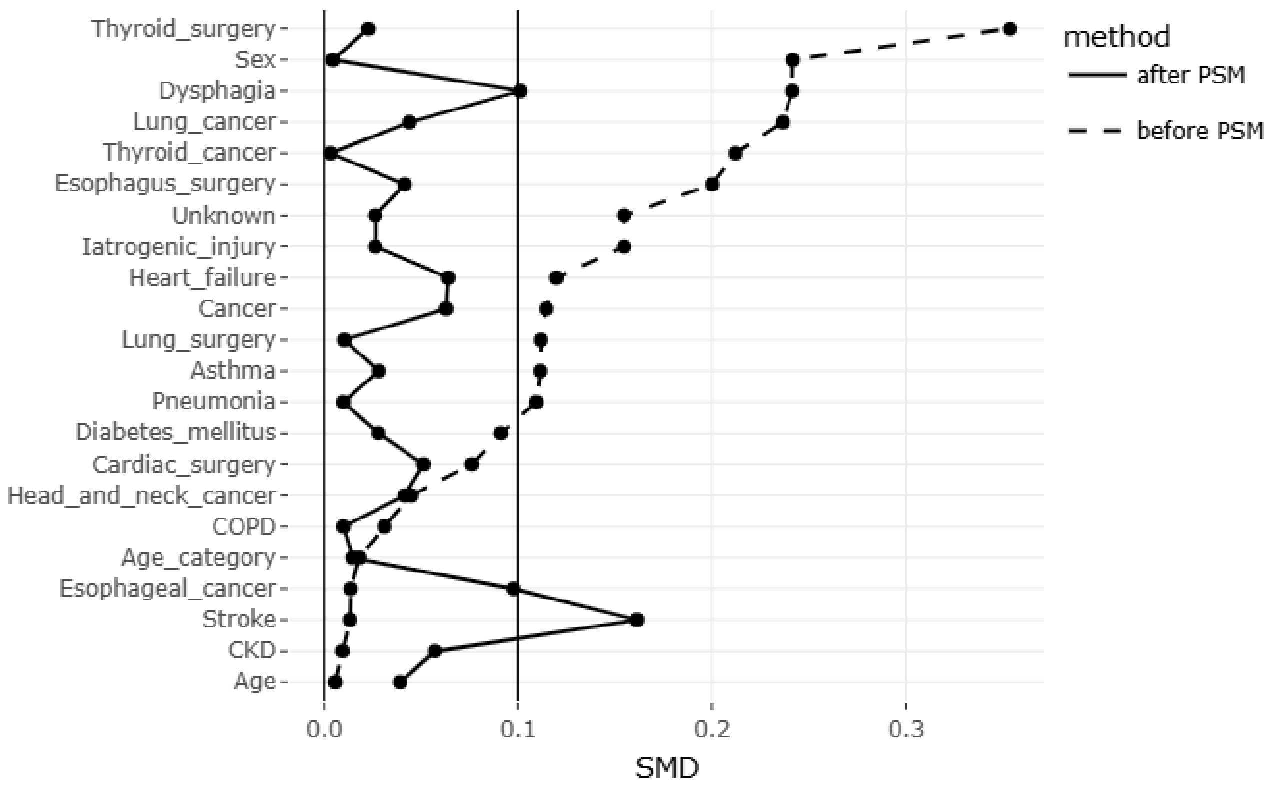

Supplement: S1 Fig — (PDF) [file pone.0352874.s001.pdf]
