# Supplementary figures and images for: Association between laryngoplasty and pneumonia incidence in patients with unilateral vocal fold paralysis: A Japanese insurance claims database study
Source: PLoS One. 2026 Jul 2;21(7):e0352874. doi: 10.1371/journal.pone.0352874 (PMC13327127; doi:10.1371/journal.pone.0352874)

**S2 Fig. Postoperative cumulative incidence of pneumonia by surgical procedure (IL and LFS).**

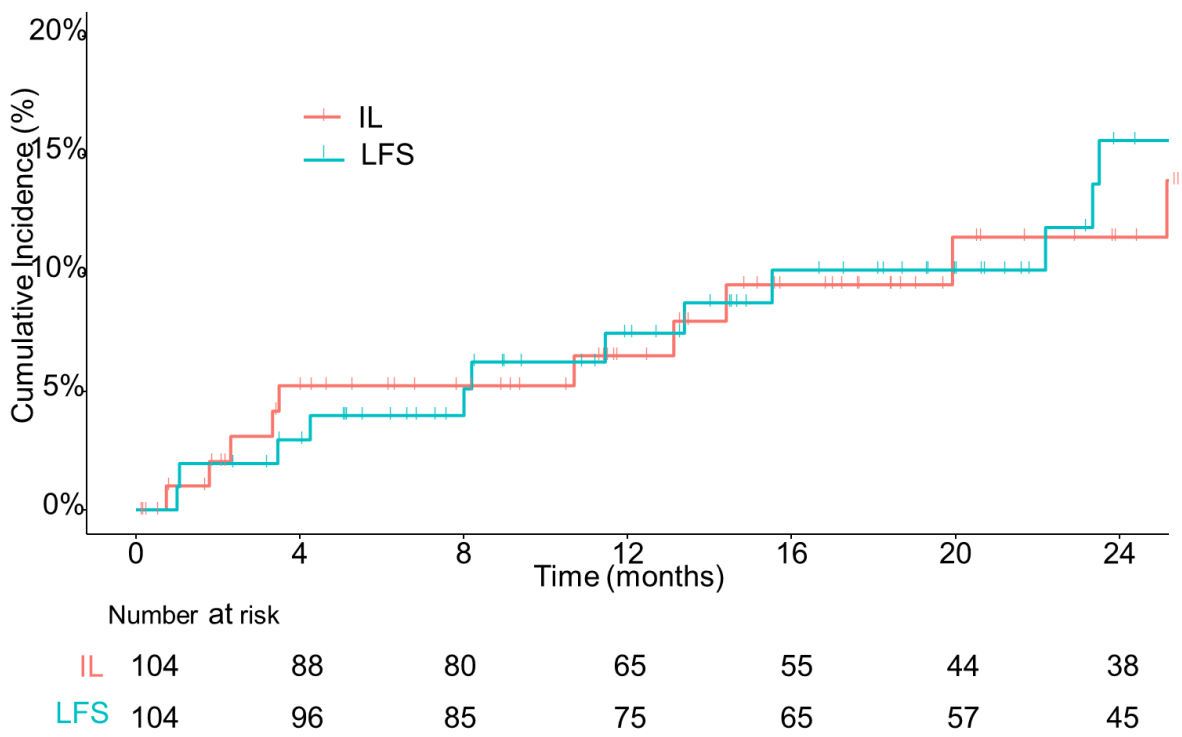

Supplement: S2 Fig — (PDF) [file pone.0352874.s002.pdf]
