# Supplementary material for: Association between laryngoplasty and pneumonia incidence in patients with unilateral vocal fold paralysis: A Japanese insurance claims database study
Source: PLoS One. 2026 Jul 2;21(7):e0352874. doi: 10.1371/journal.pone.0352874 (PMC13327127; doi:10.1371/journal.pone.0352874)
Supplement: S3 Fig — (PDF) [file pone.0352874.s003.pdf]

S3 Fig. Incidence rate of pneumonia before and after surgery in the sensitivity analysis.

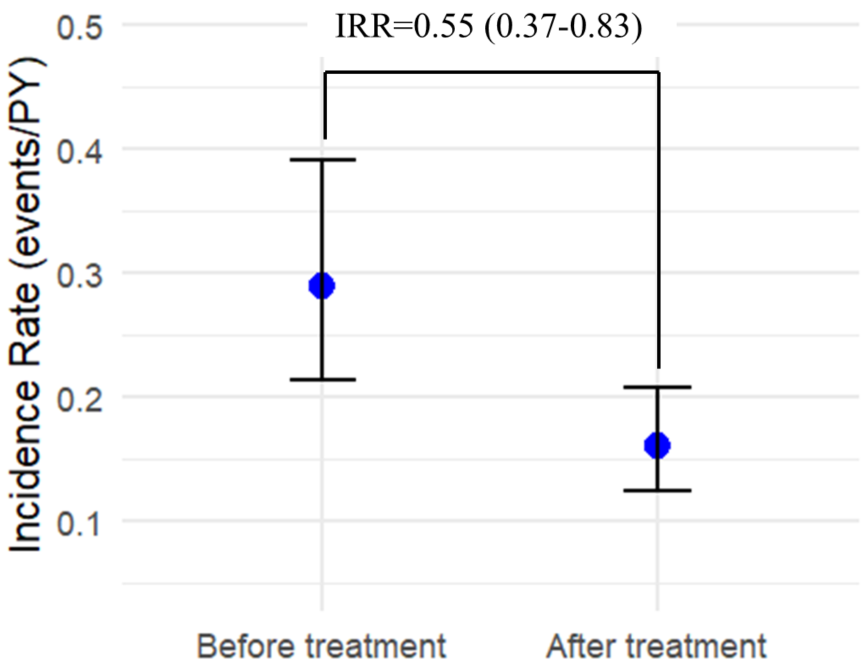

	Before treatment	After treatment
No.	163	163
Total PY	145.41	362.22
Event	42	58
IR (95% CI)	0.29 (0.21-0.39)	0.16 (0.12-0.21)
IRR (95% CI)	-	0.55 (0.37-0.83)
p value		0.004
