# Supplementary material for: Association between laryngoplasty and pneumonia incidence in patients with unilateral vocal fold paralysis: A Japanese insurance claims database study
Source: PLoS One. 2026 Jul 2;21(7):e0352874. doi: 10.1371/journal.pone.0352874 (PMC13327127; doi:10.1371/journal.pone.0352874)
Supplement: S4 Fig — (PDF) [file pone.0352874.s004.pdf]

S4 Fig. Incidence rate of pneumonia before and after surgery in the sensitivity analysis.

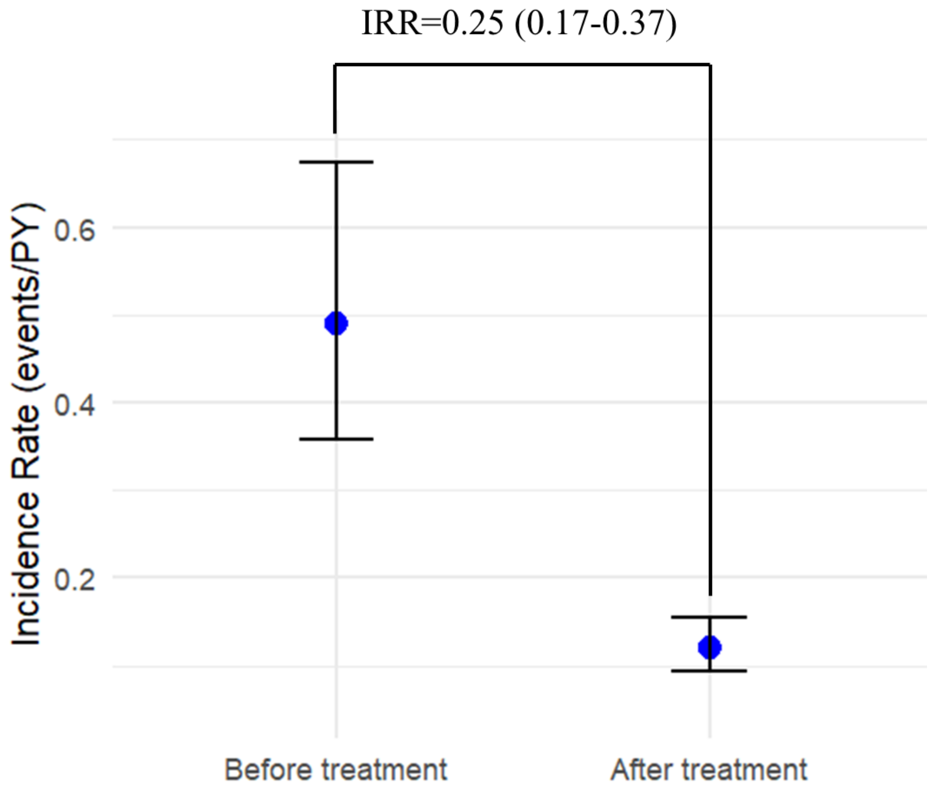

	Before treatment	After treatment
No.	177	177
Total PY	77.4	511.95
Event	67	75
IR (95% CI)	0.49 (0.35-0.67)	0.12 (0.09-0.15)
IRR (95% CI)	-	0.25 (0.17-0.37)
p value		<0.001
