# Supplementary material for: Association between laryngoplasty and pneumonia incidence in patients with unilateral vocal fold paralysis: A Japanese insurance claims database study
Source: PLoS One. 2026 Jul 2;21(7):e0352874. doi: 10.1371/journal.pone.0352874 (PMC13327127; doi:10.1371/journal.pone.0352874)
Supplement: S1 Table — (PDF) [file pone.0352874.s005.pdf]

S1 Table. ICD-10 codes and WHO-ATC codes for disease and drug definition.

Diagnosis	ICD-10 code	
**Pneumonia**		
Bacterial pneumonia	J12	Viral pneumonia, not elsewhere classified
	J13	Pneumonia due to Streptococcus pneumoniae
	J14	Pneumonia due to Hemophilus influenzae
	J15	Bacterial pneumonia, not elsewhere classified
	J16	Pneumonia due to other infectious organisms, not elsewhere classified
	J17	Pneumonia in diseases classified elsewhere
	J18	Pneumonia, unspecified organism
Aspiration pneumonia	J69	Pneumonitis due to solids and liquids
**Comorbidities**		
Asthma	J45	Asthma
Chronic kidney disease	N18	Chronic kidney disease (CKD)
Chronic obstructive pulmonary disease	J43	Emphysema
Diabetes mellitus	E10-E14	Diabetes mellitus
Heart failure	I50	Heart failure
Stroke	I63	Cerebral infarction
Head and neck cancer	C00-C14	Malignant neoplasms of lip, oral cavity and pharynx
Lung cancer	C34	Malignant neoplasm of bronchus and lung
Thyroid cancer	C73	Malignant neoplasm of thyroid gland
Esophageal cancer	C15	Malignant neoplasm of oesophagus
Dysphagia	R13	Dysphagia
**Drug definition**	**ATC code**	
**Antibiotic**		
Tetracyclines	J01A	
Amphenicols	J01B	
Beta-lactam antibacterials, penicillins	J01C	
Other beta-lactam antibacterials	J01D	
Sulfonamides and trimethoprim	J01E	
macrolides, lincosamides and streptogramins	J01F	
Aminoglycoside antibacterials	J01G	
Quinolone antibacterials	J01M	
Combinations of antibacterials	J01R	
Other antibacterials	J01X	
