# Supplementary material for: Association between laryngoplasty and pneumonia incidence in patients with unilateral vocal fold paralysis: A Japanese insurance claims database study
Source: PLoS One. 2026 Jul 2;21(7):e0352874. doi: 10.1371/journal.pone.0352874 (PMC13327127; doi:10.1371/journal.pone.0352874)
Supplement: S2 Table — (PDF) [file pone.0352874.s006.pdf]

S2 Table. Incidence rate of pneumonia before and after surgery in the subgroup analysis by surgical procedure (IL and LFS).

Treatment Procedure	IL		LFS	
	Before treatment	After treatment	Before treatment	After treatment
No.	104	104	104	104
Total PY	70.57	227.06	74.56	295.15
Event	24	36	17	37
IR (95% CI)	0.34 (0.25-0.54)	0.16 (0.12-0.24)	0.22 (0.14-0.36)	0.12 (0.08-0.16)
IRR (95% CI)	-	0.47 (0.28-0.79)	-	0.54 (0.31-0.97)
p-value	-	0.003	-	0.031
