# Supplementary material for: Association between laryngoplasty and pneumonia incidence in patients with unilateral vocal fold paralysis: A Japanese insurance claims database study
Source: PLoS One. 2026 Jul 2;21(7):e0352874. doi: 10.1371/journal.pone.0352874 (PMC13327127; doi:10.1371/journal.pone.0352874)
Supplement: S3 Table — (PDF) [file pone.0352874.s007.pdf]

S3 Table. Incidence Rate Ratio Estimation of Pneumonia Risk Factors by Poisson Regression Model

Risk factor	No.	Total PY	Pneumonia		IRRs
			Event	IR (event/PY)	(95% CI)
Age category					
18-50	466	1730.68	162	0.09	Reference
≧51	448	1328.3	417	0.31	2.87 (2.31-3.35)
Sex					
Female	466	1730.68	162	0.09	Reference
Male	448	1328.3	417	0.31	1.10 (0.93-1.31)
Cancer*					
No	526	1884.97	312	0.17	Reference
Yes	388	1174.01	267	0.23	1.12 (0.94-1.32)
Dysphagia					
No	824	2838.25	413	0.15	Reference
Yes	90	220.73	166	0.75	4.12 (3.41-4.96)
Iatrogenic injury					
No	754	2598.97	492	0.19	Reference
Yes	160	460.01	87	0.19	0.85 (0.67-1.07)

* Cancer includes head and neck cancer, lung cancer, thyroid cancer, and esophageal cancer.
Abbreviations: IR, incidence rate; PY, person-year; IRR, incidence rate ratio; CI, confidence interval.
