# Supplementary material for: Association between laryngoplasty and pneumonia incidence in patients with unilateral vocal fold paralysis: A Japanese insurance claims database study
Source: PLoS One. 2026 Jul 2;21(7):e0352874. doi: 10.1371/journal.pone.0352874 (PMC13327127; doi:10.1371/journal.pone.0352874)
Supplement: S4 Table — (PDF) [file pone.0352874.s008.pdf]

S4 Table. Pneumonia incidence estimated using Cox proportional hazards models in the propensity score–matched cohort.

Treatment	HR	95% CI	p value	Adjusted HR[a]	95% CI	p value
Non-treatment group	1.00	Reference				
Treatment group	1.40	0.94–2.09	0.098	1.35	0.90–2.01	0.149

[a]Adjusted HR estimated from a Cox model additionally adjusted for stroke and dysphagia.
