# Supplementary material for: Association between laryngoplasty and pneumonia incidence in patients with unilateral vocal fold paralysis: A Japanese insurance claims database study
Source: PLoS One. 2026 Jul 2;21(7):e0352874. doi: 10.1371/journal.pone.0352874 (PMC13327127; doi:10.1371/journal.pone.0352874)
Supplement: Attachment — Submitted filename: Comments.docx [file pone.0352874.s009.docx]

The topic is clinically important, as aspiration pneumonia remains a key complication in UVFP. The study demonstrates a significant short-term reduction in pneumonia incidence after laryngoplasty, suggesting preventive potential in high-risk groups. However, several clarifications and minor improvements in methodological description and discussion are recommended to strengthen interpretability and transparency.

Comments

It is not entirely clear whether only the first pneumonia event per patient was analyzed or if recurrent events were also included. This detail is critical for interpreting the Poisson regression results.

The manuscript should specify the list of comorbidities used in propensity score calculation (e.g., based on Charlson Comorbidity Index components or ICD-10 codes). This transparency will help readers assess covariate balance validity.

The authors mention possible immortal time bias but only partially address it. Please elaborate on the analytic approach used to minimize this bias, especially given that follow-up starts at different times relative to surgery. Indicate whether a sensitivity analysis excluding early follow-up cases altered the results.

The discussion could better articulate how laryngoplasty reduces pneumonia—beyond glottic closure restoration—by referencing improved airway clearance, cough efficacy, and swallowing biomechanics. This would contextualize the observed reduction in incidence more clearly for clinicians.

The discussion clearly presents the clinical implications of laryngoplasty but lacks integration of prior pathophysiological evidence explaining pulmonary complications following airway obstruction. Inserting the below phrase will strengthen the discussion by linking current findings with previously reported mechanisms of pulmonary complications, thereby providing a coherent physiological basis for the observed reduction in pneumonia incidence.

“Recent study demonstrated how sudden relief of upper airway obstruction can precipitate acute pulmonary compromise due to abrupt shifts in intrathoracic and vascular pressures. These findings underscore the critical importance of maintaining airway stability to prevent secondary respiratory complications [Sheikh et al., 2024, <https://doi.org/10.18231/j.ijfcm.2024.028> ]. In line with this pathophysiological understanding, the present study’s observation that laryngoplasty reduces pneumonia incidence further supports the notion that controlled and sustained restoration of airway function plays a preventive role in mitigating pulmonary morbidity.”

Implication

The discussion section misses an explicit linkage between histopathological findings and their clinical implications for patient management. Add below phrase to show practical application to the management of related condition.

“Previous research in poisoning cases identified pulmonary edema, pneumonia, and other respiratory complications as frequent findings [Jain et al., 2024 <https://pmc.ncbi.nlm.nih.gov/articles/PMC11513817/> ]. The present study may provide valuable insights for clinicians managing poisoning cases, as early recognition and prevention of pulmonary complications such as pneumonia could help reduce morbidity and mortality in similar clinical settings.”
