# Supplementary material for: Association between laryngoplasty and pneumonia incidence in patients with unilateral vocal fold paralysis: A Japanese insurance claims database study
Source: PLoS One. 2026 Jul 2;21(7):e0352874. doi: 10.1371/journal.pone.0352874 (PMC13327127; doi:10.1371/journal.pone.0352874)
Supplement: Attachment — Submitted filename: Author responce to reviewers 20251111.docx [file pone.0352874.s010.docx]

Thank you very much for taking the time to review our work. Your comments are appreciated and addressed below;

Note: the quotes from reviewers are in normal font, and our response is in navy blue font. The changes are highlighted in the revised manuscript.

**Reviewer #1:**

1. MATERIALS AND METHODS:

Kindly explain how treatments were administered for multiple pneumonia incidents. Indicate the comorbidities incorporated in the propensity score model and describe how the covariate balance was visually evaluated by using Love plots or histograms. Even though the threshold for standardized mean difference is suitable, please specify any variables that still showed minor imbalances post-matching. Indicate how absent data were addressed, if relevant. As the immortal time bias will be addressed later, please include a description in the Methods section on how your design took this into account. The pneumonia definition is solid—incorporating a reference that confirms this algorithm in the JMDC database would enhance reliability. Finally, please specify the precise R package utilized for propensity score matching.

**Response**: First, we really appreciate you taking the time to offer us your comments. We thank the reviewer for thoughtful comments about our work. We have incorporated changes that reflect the detailed suggestions you have graciously provided. We also hope that our edits and the responses we provided below satisfactorily address all the issues and concerns you and the reviewers have noted.

**Changes**:

- We now provide a list of the antibiotics used to treat multiple pneumonia incidents as the supplementary material (S1 Table) and cited in the main text (page 6, lines 119).
- All comorbidities included in the propensity score model are now specified in the manuscript (page 7, lines 154–157). We have also added a Love plot to visually evaluate covariate balance, as shown in S1 Fig. The result section of the manuscript now contains the following sentence: ‘some variables, such as stroke and dysphagia, still exceeded the 0.1 threshold (supplementary material, S1 Fig)’ (page 9, line 175-176).
- As the present study included only complete data, no imputation for missing values was required.
- To address the immortal time bias caused by differential censoring, a sensitivity analysis was conducted that excluded cases in which the post-treatment period was shorter than the pre-treatment period. The materials and methods section of the manuscript now contains the following sentence: ‘To address potential immortal time bias related to differential censoring or loss to follow-up, a sensitivity analysis was conducted by excluding cases in which the post-treatment period was shorter than the pre-treatment period’ (page 6, lines 121–124).
- The pneumonia definition algorithm used in this study has been validated in previous studies utilizing the JMDC database. The materials and methods section of the manuscript now contains the following sentence: ‘This algorithm in the JMDC database has been used in previous studies^21,24^’ (page 6, line 121).
- We now specify the R package in the Statistical Analysis section (page 7, line 137) as you suggested.

2. RESULTS:

Graphs provide valuable information but would improve with larger font sizes for the axis labels and more distinct legends. In addition to IRRs, provide 95% confidence intervals for incidence rates. In the subgroup analysis, specify sample sizes and p-values for the injection laryngoplasty and laryngeal framework surgery groups.

**Response**: We appreciate the reviewer’s constructive comment. We revised the figures to improve readability and clarity.

**Changes**:

- The font sizes of the axis labels and legends in the Kaplan–Meier plots were increased for better readability (Fig 3, S3 Fig).
- 95% confidence intervals for incidence rates were provided (Fig 4, S2 Fig, S2 Table).
- In the subgroup analysis, sample size and p-values for the injection laryngoplasty and laryngeal framework surgery groups were added. The result section of the manuscript now contains the following sentence: ‘In the subgroup analysis by surgical procedure, both IL (n= 104) and LFS (n=104) were associated with a reduction in pneumonia incidence after treatment (S2 Table). The IRR decreased from 0.34 to 0.16 for IL (IRR, 0.47; 95% CI, 0.28–0.79; p = 0.003) and from 0.22 to 0.12 for LFS (IRR, 0.54; 95% CI, 0.31–0.97; p = 0.031)’. (page 12, line 218-221).

3. DISUCUSSION :

A brief expansion on population and methodological variations that could clarify the differing hazard ratios would improve interpretation. The part regarding clinical implications could further stress whether the noted short-term decrease advocates for earlier surgical procedures or more precise selection of high-risk individuals. The limitations are thorough and straightforward; however, think about positioning the immortal-time-bias explanation earlier in this section to underscore analytic caution. Including a comment on possible supplementary functions of swallowing therapy or post-surgery care would enhance the overall clinical viewpoint.

You might think about referencing the review Heart and Lung Dysfunction Prevention Through Rehabilitation and Physical Therapy Education [ DOI: doi.org/10.47363/JCRRR/2024(5)192] to bolster more general arguments regarding how rehabilitation and the preservation of pulmonary function can enhance respiratory health. Nevertheless, exercise caution not to exaggerate its importance: it addresses general cardiopulmonary rehabilitation, excluding dysphagia or aspiration in vocal fold paralysis. Your main assertions should be supported by more detailed evidence. Utilize this as additional context, rather than a central basis.

**Response**: We appreciate this valuable suggestion. We revised the discussion section to clarify population and methodological differences, highlight the clinical implications of early surgical intervention, and expand the discussion on postoperative management and rehabilitation.

**Changes**:

- The discussion was expanded to address both population and methodological variations in order to clarify the differences in hazard ratios compared with previous reports. The discussion section of the manuscript now contains the following sentence: ‘The discrepancy between these findings may result from differences in patient populations. Particularly, the present study included patients with more severe cases who were at higher risk of developing pneumonia reflecting real-world clinical practice, as several Japanese studies have reported the therapeutic efficacy of laryngoplasty in UVFP patients with an aspiration risk^27-29^. In addition, variations in the definition of pneumonia and in the classification of surgical procedures may have led to differences in event ascertainment’ (page 14, lines 253–258).
- The clinical implication that early surgical intervention should be appropriately performed in high-risk patients was emphasized. The discussion section of the manuscript now contains the following sentence: ‘These results suggest that performing IL or LFS within approximately six months of onset may help reduce pneumonia incidence among high-risk individuals with UVFP, such as older patients and those with dysphagia or cancer, as identified in the Poisson regression analysis. Our findings emphasize the importance of appropriate patient selection and early surgical intervention for UVFP to prevent pneumonia in real-world clinical settings’ (page 18, lines 332-336).
- The rationale and interpretation of the sensitivity analysis were repositioned earlier in the discussion to improve clarity regarding immortal time bias. The discussion section of the manuscript now contains the following sentence: ‘However, censoring events such as death or disenrollment from employees’ health insurance could not be distinguished in this database. Because censoring does not occur before laryngoplasty, a decrease in pneumonia incidence observed after surgery could be influenced by immortal time bias. To address this potential bias, we performed a sensitivity analysis that excluded cases in which the post-treatment period was shorter than the pre-treatment period. The results remained consistent, indicating that the observed decrease in pneumonia incidence after surgery was robust’ (page 15, lines 268–273).
- A comment on the potential influence and supplementary functions of swallowing therapy and post-surgical care was added. The discussion section of the manuscript now contains the following sentence: ‘Furthermore, swallowing therapy, such as chin tuck, neck extension, head turn, supraglottic swallow, super-supraglottic swallow, and dietary modification has been shown to be effective for UVFP with dysphagia^1,43^ ‘ (page 17, line 307-309) ’However, the effect of injected hyaluronic acid filler is generally reported to last for only 4–6 months, after which natural absorption may lead to recurrence of aspiration^49^. Therefore, it is desirable to conduct objective evaluations such as fiberoptic endoscopic evaluation of swallowing (FEES) or video fluoroscopic swallow study (VFSS) before and after surgery to assess swallowing function and the presence of aspiration over time^50^’ (page 17-18, lines 322–326).
- Relevant literature on physical therapy approaches aimed at preserving pulmonary function was referenced. The discussion section of the manuscript now contains the following sentence: ‘In addition, pulmonary rehabilitation techniques such as straw breathing, pursued-lip breathing, and equal breathing enhance diaphragmatic activity and increase lung capacity, thereby contributing to the preservation of pulmonary function^44^. Due to the characteristics of the database in the present study, we were unable to identify the underlying causes of non-iatrogenic paralysis or determine whether patients received any rehabilitation. Therefore, to isolate the effect of laryngoplasty and minimize confounding factors from other surgical swallowing interventions, we excluded patients who underwent surgeries to improve swallowing. Appropriate postoperative care following laryngoplasty may have also contributed to the observed reduction in pneumonia incidence’ (page 17, lines 309–316).

**Reviewer #2:**

The topic is clinically important, as aspiration pneumonia remains a key complication in UVFP. The study demonstrates a significant short-term reduction in pneumonia incidence after laryngoplasty, suggesting preventive potential in high-risk groups. However, several clarifications and minor improvements in methodological description and discussion are recommended to strengthen interpretability and transparency.

**Response**: We really appreciate you taking the time to offer us your comments. We thank the reviewer for thoughtful comments about our work. We have incorporated changes that reflect the detailed suggestions you have graciously provided. We also hope that our edits and the responses we provided below satisfactorily address all the issues and concerns you and the reviewers have noted.

1. It is not entirely clear whether only the first pneumonia event per patient was analyzed or if recurrent events were also included. This detail is critical for interpreting the Poisson regression results.

**Response**: We appreciate this valuable comment. We specified that recurrent pneumonia episodes during the follow-up period were included in the analysis as count data to ensure accurate interpretation of the Poisson regression results.

**Changes**: The methods section of the manuscript now contains the following sentence: ‘Each pneumonia episode accompanied by antibiotic prescription was considered an independent recurrent event during the follow-up period, and the number of such events was analyzed as count data’ (page 6, lines 119–121).

2. The manuscript should specify the list of comorbidities used in propensity score calculation (e.g., based on Charlson Comorbidity Index components or ICD-10 codes). This transparency will help readers assess covariate balance validity.

**Response**: We appreciate this important comment. To improve transparency and reproducibility, we specified the list of comorbidities and their corresponding ICD-10 codes used in the propensity score model , which are now provided in the supplementary material (S1 Table).

**Changes**: The list of ICD-10 codes used to define comorbidities has been added. The materials and methods section of the manuscript now contains the following sentence: ‘Comorbidities were also identified according to ICD-10 codes listed in the supplementary material (S1 Table)’ (page 7, lines 136–137).

3. The authors mention possible immortal time bias but only partially address it. Please elaborate on the analytic approach used to minimize this bias, especially given that follow-up starts at different times relative to surgery. Indicate whether a sensitivity analysis excluding early follow-up cases altered the results

**Response**: We appreciate this crucial point. To address this issue, we elaborated on the analytic approach used to minimize immortal time bias and clarified the results of the corresponding sensitivity analysis. Specifically, we conducted a sensitivity analysis that excluded cases with short post-treatment follow-up periods to reduce the potential effect of immortal time bias and confirmed that the findings from this sensitivity analysis were consistent with those of the primary outcome, as shown at the end of the results section and in the supplementary material (S2 Fig).

**Changes**:

- The rationale and description of the sensitivity analysis to minimize immortal time bias was added. The materials and methods section of the manuscript now contains the following sentence: ‘To address potential immortal time bias related to differential censoring or loss to follow-up, a sensitivity analysis was conducted by excluding cases in which the post-treatment period was shorter than the pre-treatment period’ (page 6, lines 121–124). The results section of the manuscript now contains the following sentence: ‘Of the 230 patients in the treatment group, 53 were excluded due to a post-treatment period shorter than the pre-treatment period. Among the remaining 177 patients, the IRR of pneumonia after surgery was significantly lower than that before surgery (0.54; 95% CI: 0.31–0.97; p < 0.001), indicating a consistent reduction in pneumonia incidence following laryngoplasty (supplementary material, S2 Fig)’ (page 13, lines 234–238).
- The interpretation of the analysis and the robustness of the results were described. The discussion section of the manuscript now contains the following sentence: ‘Because censoring does not occur before laryngoplasty, a decrease in pneumonia incidence observed after surgery could be influenced by immortal time bias. To address this potential bias, we performed a sensitivity analysis that excluded cases in which the post-treatment period was shorter than the pre-treatment period. The results remained consistent, indicating that the observed decrease in pneumonia incidence after surgery was robust’ (page 15, lines 269–273).

4. The discussion could better articulate how laryngoplasty reduces pneumonia—beyond glottic closure restoration—by referencing improved airway clearance, cough efficacy, and swallowing biomechanics. This would contextualize the observed reduction in incidence more clearly for clinicians.

**Response**: We appreciate this insightful comment. To clarify the mechanisms through which laryngoplasty may reduce pneumonia, we expanded the discussion and incorporated relevant evidence. We referenced studies demonstrating that laryngoplasty enhances airway clearance and cough strength by improving subglottic pressure and glottic function. We additionally cited literature investigating improvements in swallowing function following laryngoplasty, providing further support for the observed reduction in pneumonia incidence.

**Changes**:

- A more detailed explanation by referencing evidence demonstrating that laryngoplasty improves airway clearance and cough efficacy was provided. The discussion section of the manuscript now contains the following sentence: ‘Watanabe et al. reported that the combination of arytenoid adduction and medialization laryngoplasty significantly improved cough peak flow in patients with UVFP^38^. This finding suggests that laryngoplasty not only strengthens cough through increased intrathoracic pressure but also helps restore airway protection^39^’ (page 16, lines 292–295).
- Literature describing improvements in swallowing function and their relevance to pneumonia prevention was added. The discussion section of the manuscript now contains the following sentence: ‘Moreover, Bruno et al. demonstrated that in UVFP patients with dysphagia, residual pressure at the upper esophageal sphincter measured by high-resolution manometry increased after laryngoplasty^40^. This finding indicates that improvement in subglottic pressure may enhance intrabolus pressure generation, playing a critical role in restoring pharyngeal clearance^29^ and reducing the risk of aspiration-related pneumonia’ (page 16, lines 295–299).

5. The discussion clearly presents the clinical implications of laryngoplasty but lacks integration of prior pathophysiological evidence explaining pulmonary complications following airway obstruction. Inserting the below phrase will strengthen the discussion by linking current findings with previously reported mechanisms of pulmonary complications, thereby providing a coherent physiological basis for the observed reduction in pneumonia incidence.“Recent study demonstrated how sudden relief of upper airway obstruction can precipitate acute pulmonary compromise due to abrupt shifts in intrathoracic and vascular pressures. These findings underscore the critical importance of maintaining airway stability to prevent secondary respiratory complications [Sheikh et al., 2024, https://doi.org/10.18231/j.ijfcm.2024.028 ]. In line with this pathophysiological understanding, the present study’s observation that laryngoplasty reduces pneumonia incidence further supports the notion that controlled and sustained restoration of airway function plays a preventive role in mitigating pulmonary morbidity.”

**Response**: We appreciate this helpful suggestion. We agreed that integrating prior pathophysiological evidence would strengthen the discussion. Accordingly, we incorporated your phrase verbatim into the discussion section to link our findings with previously reported mechanisms of pulmonary complications following airway obstruction.

**Changes**: The suggested phrase by the reviewer was inserted following the discussion of pneumonia risk factors, emphasizing how controlled and sustained restoration of airway function contributes to the prevention of pulmonary complications (page 16, lines 284–289).

6. Implication: The discussion section misses an explicit linkage between histopathological findings and their clinical implications for patient management. Add below phrase to show practical application to the management of related condition.“Previous research in poisoning cases identified pulmonary edema, pneumonia, and other respiratory complications as frequent findings [Jain et al., 2024 https://pmc.ncbi.nlm.nih.gov/articles/PMC11513817/ ]. The present study may provide valuable insights for clinicians managing poisoning cases, as early recognition and prevention of pulmonary complications such as pneumonia could help reduce morbidity and mortality in similar clinical settings.”

**Response**: We appreciate this suggestion. We carefully reviewed the proposed citation; however, because the referenced study focuses on pulmonary complications secondary to toxic exposure—an etiology distinct from airway obstruction or vocal fold paralysis—we considered it outside the scope of the present research. To maintain thematic and clinical consistency in the discussion section, we decided not to include this reference.

**Changes**: No changes were made regarding this point.
