# Supplementary material for: Association between laryngoplasty and pneumonia incidence in patients with unilateral vocal fold paralysis: A Japanese insurance claims database study
Source: PLoS One. 2026 Jul 2;21(7):e0352874. doi: 10.1371/journal.pone.0352874 (PMC13327127; doi:10.1371/journal.pone.0352874)
Supplement: Attachment — Submitted filename: Review comments.docx [file pone.0352874.s011.docx]

Review comments

Thank you for the opportunity to review this revised manuscript. The topic is clinically meaningful; however, there remain important methodological and interpretational issues that warrant clarification before the manuscript can be considered for publication. My major comments are as follows.

Major

1. The study presents three different analytical approaches:

(1) a propensity score–matched cohort analysis with HRs,

(2) a pre–post self-controlled IRR analysis among treated patients, and

(3) a Poisson regression evaluating baseline risk factors for pneumonia (Table 3).

However, the manuscript does not clearly articulate the hierarchical role of these analyses or define the primary estimand. These analytical components answer different questions (between-person vs within-person comparisons), and their relationship should be clarified. I strongly recommend explicitly designating the primary analysis and explaining how the other analyses support the main interpretation.

2. S1 Fig shows standardized mean differences before and after matching, revealing that several important covariates (e.g., age category, COPD, esophageal cancer) remain above the usual 0.1 threshold even after matching. In addition, dysphagia, one of the strongest known risk factors for pneumonia, is not appropriately adjusted for in the matched cohort analysis and does not appear to be fully balanced after matching. Given its major clinical and statistical impact on pneumonia risk, failure to adjust for dysphagia meaningfully limits interpretability of the treatment effect estimates. This point should be addressed directly, and additional adjustment or sensitivity analyses should be considered.

The authors would be better to:

- acknowledge residual imbalance and possible residual confounding,
- justify whether additional adjustment was considered, and
- summarize the final covariate balance in the main text.

This is especially important because the matched cohort HR is one of the key analyses.

**3.** Table 3 evaluates baseline predictors of pneumonia incidence using Poisson regression, but the aim and analytic role of this model are not well explained. The analysis is not an adjustment of the treatment effect but rather a cross-sectional incidence predictor model.

To avoid reader confusion, I recommend:

- clearly stating the purpose of Table 3, or
- moving Table 3 to the Supplementary materials if it is not central to the study’s conclusions.

**4. Fig4 and** S2 Fig shows a reduced IRR after surgery, but this finding is highly susceptible to confounding by indication and event-dependent exposure. Pneumonia occurring shortly before laryngoplasty often represents the clinical deterioration that motivates surgery, inflating the preoperative incidence rate and biasing the IRR downward.

I recommend a sensitivity analysis that:

- excludes pneumonia events (and person-time) occurring in the a couple of months immediately prior to surgery, or
- treats this period as a separate "high-risk" window.

This is consistent with practices in self-controlled designs where event-dependent exposure is a risk.

The manuscript should also incorporate an expanded discussion of time-varying confounding and reverse causation.

**5.** The manuscript should address the known limitations of using claims-based pneumonia diagnoses in Japanese databases (JMDC). Although DPC-based inpatient pneumonia codes (J12–J18) have shown reasonably high PPV (approximately 70–90%) in validation studies, the accuracy of pneumonia coding in broader claims datasets such as JMDC or NDB has not been systematically validated. Aspiration pneumonia (J69) in particular is known to have lower PPV (around 60–75%).

Given these limitations, the authors should explicitly acknowledge the possibility of outcome misclassification, especially because pneumonia diagnosis is the primary endpoint.

6. Lack of adjustment for preoperative pneumonia severity

The manuscript reports the percentage of patients with pneumonia before surgery but does not address the severity of preoperative pneumonia, including: pneumonia frequency (single vs recurrent episodes), oxygen requirement, hospitalization status, use of systemic antibiotics, laboratory or imaging indicators of severity.

These factors may strongly influence both the likelihood of receiving surgery and the postoperative pneumonia risk, yet they were not included in the matching algorithm or adjusted for in the analyses.

As a result, unmeasured confounding related to preoperative disease severity is likely, and the authors should discuss this limitation and consider whether additional adjustment or stratified analyses are feasible.

**7.** The manuscript would benefit from a strengthened Limitations section, including:

- residual confounding due to imperfect PS matching,
- susceptibility of pre–post IRR to time-varying confounding and confounding by indication,
- risk of event-dependent exposure, and
- outcome misclassification inherent to claims data.

**Minor**

Fig 1 and 2 are not available in the manuscript.
