# Supplementary material for: Association between laryngoplasty and pneumonia incidence in patients with unilateral vocal fold paralysis: A Japanese insurance claims database study
Source: PLoS One. 2026 Jul 2;21(7):e0352874. doi: 10.1371/journal.pone.0352874 (PMC13327127; doi:10.1371/journal.pone.0352874)
Supplement: Attachment — Submitted filename: Author responce to reviewers 20260113ver2.docx [file pone.0352874.s012.docx]

Thank you very much for taking the time to review our work. Your comments are appreciated and addressed below;

Note: the quotes from reviewers are in normal font, and our response is in navy blue font. The changes are highlighted in the revised manuscript.

**Reviewer #3:**

I was not involved in the first round of peer review and have joined the review process from this revision. Therefore, I focused my evaluation on whether the issues raised in the previous round have been appropriately addressed in the current revision. From this perspective, I find that the authors have thoroughly considered the previous reviewer’s comments and have incorporated the requested revisions appropriately.

On the other hand, the Discussion section has become quite long and redundant, which I personally find a bit concerning. However, this appears to be a result of the authors’ sincere efforts to address the prior comments. Whether further condensation of the Discussion is necessary may be best left to the editor’s discretion.

**Response**:

We thank the reviewer for the careful evaluation of the revised manuscript and for the positive assessment that the concerns raised in the previous round were appropriately addressed.

We also appreciate the comment regarding the length and potential redundancy of the Discussion section. In the revised version, we have carefully reviewed the Discussion and condensed overlapping statements where possible. Please note that we included additional discussion in the revised manuscript, as requested in the current review.

**Reviewer #4:**

Thank you for the opportunity to review this revised manuscript. The topic is clinically meaningful; however, there remain important methodological and interpretational issues that warrant clarification before the manuscript can be considered for publication. My major comments are as follows.

**Response**: We really appreciate you taking the time to offer us your comments. We thank the reviewer for thoughtful comments about our work. We have incorporated changes that reflect the detailed suggestions you have graciously provided. We also hope that our edits and the responses we provided below satisfactorily address all the issues and concerns you and the reviewers have noted.

**Major**

1. The study presents three different analytical approaches:

(1) a propensity score–matched cohort analysis with HRs,

(2) a pre–post self-controlled IRR analysis among treated patients, and

(3) a Poisson regression evaluating baseline risk factors for pneumonia (Table 3).

However, the manuscript does not clearly articulate the hierarchical role of these analyses or define the primary estimand. These analytical components answer different questions (between-person vs within-person comparisons), and their relationship should be clarified. I strongly recommend explicitly designating the primary analysis and explaining how the other analyses support the main interpretation.

**Response**: We appreciate this important comment. We agree that the initial version of the manuscript did not sufficiently clarify the hierarchical role of the different analytical approaches or explicitly define the primary estimand.

To address this concern, we have revised the materials and methods section to clearly designate the pre–post self-controlled incidence rate ratio (IRR) analysis among treated patients as the primary analysis. This analysis estimates within-person changes in pneumonia incidence before and after laryngoplasty and was designed to provide a causal estimate of the treatment effect while minimizing confounding by time-invariant individual characteristics.

The propensity score–matched cohort analysis comparing the cumulative incidence of pneumonia between the treatment and non-treatment groups is now explicitly described as a secondary analysis, intended to complement the primary analysis by assessing potential differences in pneumonia risk at the population level.

In addition, the Poisson regression model evaluating baseline predictors of pneumonia has been repositioned as an exploratory analysis, aimed at identifying baseline risk factors and vulnerable subpopulations among patients with UVFP, rather than serving as an adjustment model for treatment effect estimation.

These revisions clarify the distinct objectives of each analytical approach and their respective roles in supporting the overall interpretation of the study findings.

**Changes**: The materials and methods section of the manuscript now contains the following sentence:

- ‘As the primary analysis, we conducted a self-controlled analysis within the treatment group to compare the incidence of pneumonia before and after laryngoplasty. This analysis aimed to evaluate within-person changes in pneumonia incidence associated with the period before and after surgery, thereby minimizing confounding by time-invariant individual characteristics.’ (page 6, lines 119–122).
- ‘As the secondary analysis, we compared the cumulative incidence of pneumonia between the treatment group and the non-treatment group, with follow-up starting from a predefined index date, defined as the onset of UVFP. This analysis aimed to assess potential differences in pneumonia risk at the population level between patients who underwent laryngoplasty and those who did not’ (page 6-7, lines 126–130).
- ‘In addition, as an exploratory analysis, we examined baseline predictors of pneumonia in the overall cohort. This analysis aimed to identify vulnerable subpopulations at higher risk of pneumonia among patients with UVFP and to inform appropriate patient selection for future preventive interventions’ (page 7, lines 130–133).

2. S1 Fig shows standardized mean differences before and after matching, revealing that several important covariates (e.g., age category, COPD, esophageal cancer) remain above the usual 0.1 threshold even after matching. In addition, dysphagia, one of the strongest known risk factors for pneumonia, is not appropriately adjusted for in the matched cohort analysis and does not appear to be fully balanced after matching. Given its major clinical and statistical impact on pneumonia risk, failure to adjust for dysphagia meaningfully limits interpretability of the treatment effect estimates. This point should be addressed directly, and additional adjustment or sensitivity analyses should be considered.

The authors would be better to:

acknowledge residual imbalance and possible residual confounding,

justify whether additional adjustment was considered, and

summarize the final covariate balance in the main text.

This is especially important because the matched cohort HR is one of the key analyses.

**Response**: We thank the reviewer for this valuable comment. We agree that several important covariates associated with pneumonia risk were not fully balanced after propensity score matching and that additional adjustment for these variables was warranted.

To address this concern, we conducted a sensitivity analysis using multivariable Cox proportional hazards models that additionally included the covariates with standardized mean differences greater than 0.1 after matching, specifically stroke and dysphagia. In the results section, we now explicitly acknowledge the presence of residual imbalance and potential residual confounding and report the adjusted hazard ratios derived from this analysis. Importantly, the adjusted hazard ratio for pneumonia was similar in direction and magnitude to that observed in the propensity score–matched secondary analysis, supporting the interpretability of the between-group comparison of pneumonia incidence.

**Changes**:

- The materials and methods section of the manuscript now contains the following sentence: ‘Furthermore, if any covariates remained insufficiently balanced after PS matching, we performed additional adjustment using multivariable Cox proportional hazards models including these covariates, as recommended in previous studies to mitigate residual confounding in matched observational studies[27, 28]’ (page 8, lines 163–166).
- The results section of the manuscript now contains the following sentence: ‘Furthermore, in multivariable Cox proportional hazards models that additionally adjusted for stroke and dysphagia, which remained imbalanced after PS matching (SMD >0.1), the treatment group tended to have a higher incidence of pneumonia compared with the non-treatment group; however, this difference was not statistically significant (adjusted HR, 1.35; 95% CI, 0.90–2.01; p = 0.149) (S4 Table)’ (page 14, lines 265–269).
- The discussion section of the manuscript now contains the following sentence: ‘As a sensitivity analysis, we applied a double-adjustment approach using multivariable Cox proportional hazards models that additionally included these covariates. Although the direction of the hazard ratio for pneumonia was consistent with the results of the secondary analysis, residual confounding cannot be fully excluded’ (page 20, lines 390–393).

3. Table 3 evaluates baseline predictors of pneumonia incidence using Poisson regression, but the aim and analytic role of this model are not well explained. The analysis is not an adjustment of the treatment effect but rather a cross-sectional incidence predictor model. To avoid reader confusion, I recommend:

・clearly stating the purpose of Table 3, or

・moving Table 3 to the Supplementary materials if it is not central to the study’s conclusions.

**Response**: We appreciate this helpful comment. We agree that the Poisson regression analysis evaluating baseline predictors of pneumonia was not intended to estimate the treatment effect and may have caused confusion if presented alongside the primary and secondary analyses. To clarify its analytic role, we have repositioned this analysis as an exploratory analysis and moved the corresponding table to the Supplementary Materials. This analysis is now presented solely to describe baseline risk factors for pneumonia among patients with UVFP, rather than as an adjustment or supportive analysis of the treatment effect.

**Changes**:

- The Poisson regression analysis of baseline predictors of pneumonia has been moved from the main text to the Supplementary Materials (S3 Table). (page 13, line 247).

4. Fig4 and S2 Fig shows a reduced IRR after surgery, but this finding is highly susceptible to confounding by indication and event-dependent exposure. Pneumonia occurring shortly before laryngoplasty often represents the clinical deterioration that motivates surgery, inflating the preoperative incidence rate and biasing the IRR downward. I recommend a sensitivity analysis that:

・excludes pneumonia events (and person-time) occurring in the a couple of months immediately prior to surgery, or

・treats this period as a separate "high-risk" window.

This is consistent with practices in self-controlled designs where event-dependent exposure is a risk. The manuscript should also incorporate an expanded discussion of time-varying confounding and reverse causation.

**Response**: We appreciate this insightful comment. We agree that the observed reduction in the incidence rate ratio (IRR) in the pre–post comparison may be susceptible to event-dependent exposure, particularly if pneumonia events occurring shortly before surgery influenced the clinical decision to perform laryngoplasty. Such a mechanism could bias the preoperative pneumonia incidence upward and lead to an overestimation of the apparent treatment effect.

To address this concern, we conducted an additional sensitivity analysis in which pneumonia events occurring within the 2 months immediately preceding laryngoplasty, as well as the corresponding person-time during this period, were excluded as the reviewer suggested. In addition, we expanded the discussion section to explicitly consider the possibility that the observed reduction in IRR in the primary analysis may have been influenced by time-varying confounding and reverse causation.

Importantly, even under this more conservative assumption, the incidence of pneumonia after laryngoplasty remained lower than that before surgery, demonstrating a consistent direction of association. We believe that these additional analyses and the expanded discussion strengthen the robustness and interpretability of the self-controlled analysis.

**Changes**:

- The material and methods section of the manuscript now contains the following sentence: ‘In the primary analysis, the self-controlled comparison of pneumonia incidence before and after laryngoplasty may be susceptible to confounding if pneumonia events occurring shortly before surgery influence the indication for laryngoplasty. To address this concern, we conducted a sensitivity analysis in which pneumonia events occurring within the 2 months immediately preceding laryngoplasty, as well as the corresponding person-time during this period, were excluded from the analysis’ (page 8, lines 156–160).
- The results section of the manuscript now contains the following sentence: ‘Of the 230 patients in the treatment group, 67 were excluded from the analysis because their preoperative observation period was shorter than 2 months. Among the remaining 163 patients, comparison of the period from the index date to 2 months before surgery with the postoperative observation period showed that the incidence of pneumonia was significantly lower after laryngoplasty than before surgery (IRR 0.55; 95% CI, 0.37–0.83; p = 0.004) (S3 Fig)’ (page 14, lines257–261).
- The discussion section of the manuscript now contains the following sentence: ‘Although the self-controlled analysis in this study minimizes time-invariant confounding through within-person comparisons, it may remain susceptible to time-varying confounding and reverse causation. In particular, pneumonia episodes occurring shortly before laryngoplasty may influence the decision to perform surgery, resulting in event-dependent exposure and a potential overestimation of preoperative pneumonia incidence. To address this concern, we conducted a sensitivity analysis excluding pneumonia events occurring within the 2 months immediately preceding laryngoplasty, as well as the corresponding person-time. In this analysis, the incidence of pneumonia after surgery remained lower than that before surgery; however, the magnitude of the reduction was attenuated, with the IRR increasing from 0.36 in the primary analysis to 0.55. This result suggests that treatment effect estimates using the time of surgery as the reference point may be partially influenced by time-varying confounding related to perioperative events’ (page 16, lines 297–306).
- The discussion section of the manuscript now contains the following sentence: ‘Taken together, these sensitivity analyses conducted under different assumptions consistently showed a lower incidence of pneumonia after surgery, suggesting that the primary findings of this study are robust and not driven by specific time-related biases, including time-varying confounding or immortal time bias’ (page 16, lines 311–314).

5. The manuscript should address the known limitations of using claims-based pneumonia diagnoses in Japanese databases (JMDC). Although DPC-based inpatient pneumonia codes (J12–J18) have shown reasonably high PPV (approximately 70–90%) in validation studies, the accuracy of pneumonia coding in broader claims datasets such as JMDC or NDB has not been systematically validated. Aspiration pneumonia (J69) in particular is known to have lower PPV (around 60–75%).

Given these limitations, the authors should explicitly acknowledge the possibility of outcome misclassification, especially because pneumonia diagnosis is the primary endpoint.

**Response**: The reviewer has raised an important point here. We agree that, at present, the diagnostic accuracy of pneumonia coding in large claims databases such as JMDC or the National Database has not been fully validated. In this study, pneumonia outcomes were identified using an algorithm that combined diagnostic codes with records of systemic antibiotic prescriptions; however, we acknowledge that outcome misclassification may still have occurred despite this approach. In response to this concern, we have strengthened the Limitations section to explicitly acknowledge the potential for misclassification of pneumonia outcomes inherent to claims-based data.

**Changes**: The discussion section of the manuscript now contains the following sentence: ‘With respect to the outcome definition, pneumonia was identified using an algorithm based on diagnostic codes combined with systemic antibiotic prescriptions; however, this algorithm has not been fully validated in the JMDC database, and the possibility of outcome misclassification cannot be excluded’ (page 19, lines 382–384).

6. Lack of adjustment for preoperative pneumonia severity

The manuscript reports the percentage of patients with pneumonia before surgery but does not address the severity of preoperative pneumonia, including: pneumonia frequency (single vs recurrent episodes), oxygen requirement, hospitalization status, use of systemic antibiotics, laboratory or imaging indicators of severity.

These factors may strongly influence both the likelihood of receiving surgery and the postoperative pneumonia risk, yet they were not included in the matching algorithm or adjusted for in the analyses.

As a result, unmeasured confounding related to preoperative disease severity is likely, and the authors should discuss this limitation and consider whether additional adjustment or stratified analyses are feasible.

**Response**: We thank the reviewer for this important comment. We agree that the lack of detailed clinical information is a major limitation of observational studies using claims databases. Although pneumonia cases in this study were identified in conjunction with records of systemic antibiotic use, detailed information on disease severity—such as the severity of pneumonia episodes, oxygen requirement, hospitalization status, or objective measures of swallowing dysfunction—was not available.

As a result, unmeasured confounding related to disease severity may remain, and patients at higher baseline risk of pneumonia may have been preferentially selected for laryngoplasty. We also acknowledge the reviewer’s concern that the observed reduction in pneumonia incidence after surgery could, in part, reflect regression to the mean rather than a pure treatment effect.

We have addressed these concerns by explicitly strengthening the limitations section. However, additional stratified analyses or further adjustments based on disease severity were not feasible because the relevant clinical indicators were not captured in the claims data. We therefore recognize this as an inherent limitation of the data source and interpret our findings with appropriate caution.

**Changes**: The discussion section of the manuscript now contains the following sentence: ‘Second, detailed clinical information, such as the severity of UVFP or pneumonia, was not available in the claims database. These factors are likely to strongly influence the likelihood of undergoing laryngoplasty, and the presence of unmeasured confounding may have resulted in a preferential selection of more severe patients into the treatment group. Consequently, the observed reduction in pneumonia incidence may, in part, reflect regression to the mean rather than a pure treatment effect.’ (page 19-20, lines 384–389).

7. The manuscript would benefit from a strengthened Limitations section, including:

residual confounding due to imperfect PS matching, susceptibility of pre–post IRR to time-varying confounding and confounding by indication, risk of event-dependent exposure, and outcome misclassification inherent to claims data.

**Response**: We appreciate this comprehensive comment. We agree that a strengthened and more explicit Limitations section was warranted to appropriately contextualize our findings.

In response, we have substantially revised and expanded the Limitations section to explicitly incorporate the issues raised in the major comments. Specifically, we now address residual confounding due to imperfect propensity score matching and unmeasured disease severity (Major Comments 2 and 6), susceptibility of the pre–post self-controlled analysis to time-varying confounding and confounding by indication, including event-dependent exposure and reverse causation (Major Comment 4), and potential outcome misclassification inherent to claims-based data (Major Comment 5). These limitations are discussed together with the corresponding sensitivity analyses to provide a transparent and balanced interpretation of the results.

We believe that these revisions comprehensively summarize the methodological limitations highlighted by the reviewer and appropriately frame the robustness and interpretability of our findings.

**Minor**

Fig 1 and 2 are not available in the manuscript.

**Response**: Thank you for pointing this out. We apologize for the oversight. Figures 1 and 2 will be carefully checked and uploaded correctly at the time of resubmission.
