# Supplementary material for: Association between laryngoplasty and pneumonia incidence in patients with unilateral vocal fold paralysis: A Japanese insurance claims database study
Source: PLoS One. 2026 Jul 2;21(7):e0352874. doi: 10.1371/journal.pone.0352874 (PMC13327127; doi:10.1371/journal.pone.0352874)
Supplement: Attachment — Submitted filename: Author responce to reviewers 20260321.docx [file pone.0352874.s013.docx]

Thank you very much for taking the time to review our work. Your comments are appreciated and addressed below;

Note: the quotes from reviewers are in normal font, and our response is in navy blue font. The changes are highlighted in the revised manuscript.

**Reviewer #1:**

A significant clinical topic about bladder sensation recovery after childbirth is addressed in this updated publication, which also offers helpful service-level data. A service evaluation is a suitable study design, and the results generally corroborate the key findings. However, their reliability and generalizability are limited by their reliance on subjective patient-reported improvement and their small sample size, which should be made clearer.

**Response**:

We appreciate you taking the time to review our work, again. However, we are afraid that the comment seems to be about a different study (bladder sensation recovery after childbirth), which is unrelated to our manuscript on laryngoplasty and pneumonia incidence in UVFP.

**Reviewer #4:**

The authors state in their response that they intend to redefine the self-controlled analysis as the primary analysis. While this represents a reasonable attempt to address unmeasured time-invariant confounding, several critical methodological and reporting issues must be resolved. These issues primarily concern (1) the validity of self control study design assumptions in the present clinical context, (2) inconsistency in study presentation, and (3) discordant findings across analytical frameworks.

**Response**: We sincerely thank the reviewer for the thorough and methodologically insightful comments. In response to these concerns, we substantially revised the analytical framework and interpretation of our findings. Importantly, we changed the title from “Reduction in Pneumonia Incidence…” to “Association Between Laryngoplasty and Pneumonia Incidence…” to avoid causal or directional implications not fully supported by the data. We also repositioned the matched cohort analysis as the primary analysis and the self-controlled analysis as secondary, and revised the Abstract, Results, Discussion and Conclusions to explicitly acknowledge that the direction of association differed depending on the analytical framework applied.

1. **Appropriateness of self control study design as the Primary Analytical Framework**

Self-controlled designs, including self control study design, rely on several key assumptions. It is currently unclear whether these assumptions are satisfied in the present study.

1.1 Assumption of event-independent exposure

A fundamental assumption of self control study design is that the occurrence of the outcome does not influence the probability or timing of exposure. In this study, pneumonia—particularly recurrent or severe pneumonia—is a major clinical indication for laryngoplasty. Consequently, pneumonia events occurring prior to surgery are likely to increase the probability of subsequent exposure (i.e., surgery). This violates the assumption of event-independent exposure and introduces event-dependent exposure (confounding by indication). Unless explicitly addressed (for example, by excluding or separately modeling a pre-exposure high-risk window), this violation may substantially bias self control study design estimates.

**Response**: We thank the reviewer for this important comment. We agree that pneumonia occurring prior to surgery may influence the timing or indication for laryngoplasty, introducing potential event-dependent exposure. To address this concern, we conducted a sensitivity analysis excluding pneumonia events occurring within the 2 months immediately preceding surgery, along with the corresponding person-time. Although the magnitude of the reduction was attenuated (IRR increased from 0.36 to 0.55), the direction of association remained unchanged.

We have clarified this issue in the Discussion and explicitly acknowledged that violation of the event-independent exposure assumption cannot be fully excluded; therefore, the self-controlled findings should be interpreted cautiously.

**Changes**:

- The materials and methods section of the manuscript now contains the following sentence: ‘The self-controlled comparison of pneumonia incidence before and after laryngoplasty may be susceptible to confounding if pneumonia events occurring shortly before surgery influence the indication for laryngoplasty. Therefore, as a sensitivity analysis, we excluded pneumonia events occurring within the 2 months immediately preceding laryngoplasty, along with the corresponding person-time.’ (page 8, lines 155–159).
- The results section of the manuscript now contains the following sentence: ‘Among the remaining 163 patients, comparison of the period from the index date to 2 months before surgery with the postoperative observation period showed that the incidence of pneumonia was significantly lower after laryngoplasty than before surgery (IRR 0.55; 95% CI, 0.37–0.83; p = 0.004) (S3 Fig).’ (page 14, lines 257–259).
- The discussion section of the manuscript now contains the following sentence: ‘Regarding the self-controlled analysis, pneumonia episodes occurring shortly before laryngoplasty may influence the decision to perform surgery, resulting in event-dependent exposure and a potential overestimation of preoperative pneumonia incidence. A sensitivity analysis excluding pneumonia events within the 2 months immediately preceding surgery attenuated the magnitude of the reduction (IRR increased from 0.36 to 0.55), suggesting that the observed effect may partially reflect perioperative time-related biases.’ (page 16, lines 294–299).
  1.2 Assumption of stable baseline risk

self control study design further assumes that baseline risk within individuals is either constant over time or adequately controlled through time-varying covariates. In the present study, several strong time-varying factors—such as progression of dysphagia, increasing frailty, perioperative care, and changes in clinical surveillance—are likely to differ systematically between pre- and post-surgical periods. These factors are not fully accounted for and may confound within-person comparisons.

**Response**: We thank the reviewer for this important point. We acknowledge that several time-varying factors, such as progression of dysphagia, frailty, perioperative care, and changes in clinical surveillance, may differ systematically between pre- and post-surgical periods. Due to the nature of the claims database, these clinical variables were not available, and changes in baseline risk over time could not be directly accounted for.

In response, we have strengthened the limitations section to explicitly acknowledge the potential influence of time-varying confounding and the need to interpret the self-controlled findings with caution.

**Changes**:

- The discussion section of the manuscript now contains the following sentence: ‘In addition, several strong time-varying factors—such as progression of dysphagia, increasing frailty, and changes in clinical surveillance—are likely to differ systematically between the pre- and post-surgical periods. Because these factors were not available in the present database, the observed reduction in pneumonia incidence before and after laryngoplasty should be interpreted with caution.’ (page 16, lines 299–302).
- ‘Third, self-controlled analysis may be susceptible to time-varying confounding and event-dependent exposure. Pneumonia episodes occurring shortly before surgery may have influenced the indication for laryngoplasty and patients’ clinical status may have differed systematically between the pre- and post-surgical periods.’ (page 19, lines 364–367).

1.3 Conclusion regarding self control study design validity

Given the likely violation of key self control study design assumptions, particularly event-dependent exposure, the manuscript should either: provide a detailed justification demonstrating that self control study design assumptions are reasonably met, or clearly acknowledge that self control study design estimates may be biased and interpret them as descriptive rather than causal. Without such clarification, using self control study design as the primary analysis is difficult to justify.

**Response**: We appreciate the reviewer’s important comment. Although we performed sensitivity analyses to mitigate potential event-dependent exposure, we agree that violation of key assumptions of the self-controlled design cannot be fully excluded. In response, we have repositioned the matched cohort analysis as the primary analysis and treated the self-controlled analysis as secondary. We have also revised the manuscript to interpret the self-controlled findings as descriptive rather than causal and to explicitly acknowledge their susceptibility to bias.

**Changes**:

- The introduction section of the manuscript now contains the following sentence: ‘Understanding the clinical outcomes of laryngoplasty may help determine the indications for surgical treatment in the management of UVFP. In this study, we conducted a cross-institutional analysis using a Japanese insurance claims database to evaluate the association between laryngoplasty and pneumonia incidence in patients with UVFP.’ (page 4, lines 71–74)
- The material and methods section of the manuscript now contains the following sentence: ‘As the primary analysis, we compared the cumulative incidence of pneumonia between the treatment group and the non-treatment group, with follow-up starting from a predefined index date, defined as the onset of UVFP.’ (page 6, lines 117–119) ‘As the secondary analysis, we conducted a self-controlled analysis within the treatment group to compare the incidence of pneumonia before and after laryngoplasty.’ (page 6, lines 120–122)
- The discussion section of the manuscript now contains the following sentence: ‘These limitations may have biased within-person comparisons. As described previously, the observed reduction in pneumonia incidence after surgery should therefore be interpreted with caution.’ (page 19, lines 367-369).

**2. Implications for Baseline Characteristics (Table 1) and Study Design (Figure 1)**

If the authors define the self-controlled analysis as the primary analytical framework, the presentation of baseline characteristics and study design must be revised accordingly.

2.1 Inconsistency of the current Table 1

Table 1 currently presents baseline characteristics comparing patients who did and did not undergo surgery. This structure is appropriate for a between-person cohort analysis but not for a self-controlled study, where comparisons are made within individuals. In self control study design, between-group baseline differences are, by design, irrelevant to the primary comparison.

**Response**: We thank the reviewer for this comment. As noted above, we have revised the manuscript to designate the matched cohort analysis as the primary analytical framework. Accordingly, the presentation of baseline characteristics comparing the treatment and non-treatment groups in Table 1 is consistent with the primary between-group design. We have clarified this structure throughout the manuscript to ensure alignment between the analytical framework and the presentation of baseline characteristics.

2.2 Recommended revision of Table 1

If self control study design is the primary analysis, Table 1 should instead describe only the population included in the self-controlled analysis, namely patients who underwent surgery. Appropriate baseline information may include: demographics of operated patients, prevalence of dysphagia and other comorbidities, frequency and severity of pneumonia prior to surgery (e.g., recurrent episodes, oxygen requirement), timing of surgery relative to prior pneumonia events. Similarly, Figure 1 (study flow diagram) should be revised to clearly reflect a self-controlled analytical framework rather than a between-group cohort comparison.

**Response**: We appreciate the reviewer’s suggestion. As described above, we have revised the analytical framework to designate the matched cohort analysis as the primary analysis. Therefore, Table 1 appropriately presents baseline characteristics comparing the treatment and non-treatment groups, and Figure 1 reflects the between-group cohort design. We have revised the manuscript to clearly describe this structure to avoid ambiguity regarding the primary analytical framework.

2.3 Overall recommendation regarding study presentation

While adopting a self-controlled design may help address unmeasured time-invariant confounding, the current study context raises serious concerns regarding the validity of self control study design assumptions, particularly because pneumonia is a major indication for surgery. If the authors wish to proceed with self control study design as the primary analysis, they must: explicitly justify the appropriateness of self control study design assumptions, address event-dependent exposure and time-varying confounding, and revise figures and baseline tables to align with a self-controlled analytic framework. Without these revisions, self control study design results should be interpreted cautiously and may be more appropriately positioned as a secondary or sensitivity analysis.

**Response**: We thank the reviewer for this comprehensive recommendation. In response, we have repositioned the matched cohort analysis as the primary analysis and treated the self-controlled analysis as secondary. We have also revised the Abstract, Methods and Discussion to explicitly acknowledge the susceptibility of the self-controlled design to event-dependent exposure and time-varying confounding, and to interpret these findings cautiously. We believe these revisions align the study presentation with the reviewer’s recommendations and clarify the analytical hierarchy of the manuscript.

**Changes**:

- The abstract section of the manuscript now contains the following sentence: ‘The primary analysis compared the cumulative incidence of pneumonia between treatment and non-treatment groups. The secondary analysis employed a self-controlled design to compare pneumonia incidence before and after laryngoplasty within treated patients.’ (page 2, lines 37–40)
- The material and methods section of the manuscript now contains the following sentence: ‘As the primary analysis, we compared the cumulative incidence of pneumonia between the treatment group and the non-treatment group, with follow-up starting from a predefined index date, defined as the onset of UVFP.’ (page 6, lines 117–119) ‘As the secondary analysis, we conducted a self-controlled analysis within the treatment group to compare the incidence of pneumonia before and after laryngoplasty.’ (page 6, lines 120–122)
- The discussion section of the manuscript now contains the following sentence: ‘Thus, our study adds to the existing literature by examining the relationship between laryngoplasty and pneumonia incidence using multiple analytical approaches within a large insurance database.’ (page 15, lines 286-288)
- The discussion section of the manuscript now contains the following sentence: ‘Regarding the self-controlled analysis, pneumonia episodes occurring shortly before laryngoplasty may influence the decision to perform surgery, resulting in event-dependent exposure and a potential overestimation of preoperative pneumonia incidence. A sensitivity analysis excluding pneumonia events within the 2 months immediately preceding surgery attenuated the magnitude of the reduction (IRR increased from 0.36 to 0.55), suggesting that the observed effect may partially reflect perioperative time-related biases. In addition, several strong time-varying factors—such as progression of dysphagia, increasing frailty, and changes in clinical surveillance—are likely to differ systematically between the pre- and post-surgical periods. Because these factors were not available in the present database, the observed reduction in pneumonia incidence before and after laryngoplasty should be interpreted with caution.’ (page 16, lines 294-302)

3. **Inconsistency Between the Abstract and the Declared Primary Analysis (self control study design)**

3.1 Issues with the current Abstract

The Abstract lacks any mention of a self-controlled or within-person study design. Key methodological elements of self control study design (e.g., comparison of pre- and post-exposure periods within individuals) are not described. As a result, readers may incorrectly assume that the primary analysis is a conventional cohort or between-person comparison.

**Response**: We thank the reviewer for this important comment. In response, we have revised the Abstract to clearly describe the matched cohort analysis as the primary analysis and the self-controlled analysis as secondary. The methodological elements of the within-person comparison are now explicitly stated to ensure clarity and consistency with the analytical framework presented in the main text. We believe these revisions prevent potential misinterpretation of the study design.

**Changes**:

- The abstract section of the manuscript now contains the following sentence: ‘The primary analysis compared the cumulative incidence of pneumonia between treatment and non-treatment groups. The secondary analysis employed a self-controlled design to compare pneumonia incidence before and after laryngoplasty within treated patients.’ (page 2, lines 37–40)

3.2 Recommended revision of the Abstract

If self control study design is the primary analysis, the Abstract should explicitly state: the use of a self-controlled or self control study design, that comparisons are made within individuals before and after surgery, and the primary effect measure derived from this approach. Failure to align the Abstract with the declared primary methodology may mislead readers and does not conform to standard reporting practices for observational studies.

**Response**: We appreciate the reviewer’s recommendation. As noted above, we have revised the analytical hierarchy of the study and designated the matched cohort analysis as the primary analysis. Accordingly, the Abstract has been revised to clearly describe the primary between-group comparison and to present the self-controlled analysis as secondary. The methodological elements of both analyses are now explicitly stated to ensure alignment between the declared primary framework and the Abstract. We believe these revisions address the reviewer’s concern regarding clarity and reporting consistency.

4. **Discordant Results Between Matched Cohort and Self-Controlled Analyses**

A major concern is that the matched cohort analysis and the self-controlled analysis yield results in opposite directions: The propensity score–matched cohort analysis suggests a higher postoperative pneumonia risk among surgically treated patients compared with matched non-surgical controls. In contrast, the self-controlled analysis suggests a lower pneumonia incidence after surgery relative to the immediate preoperative period. Given this discrepancy, the study cannot support a single directional conclusion regarding the effect of surgery on pneumonia risk. At a minimum, the findings indicate that postoperative pneumonia risk is highly sensitive to study design and analytical assumptions. Accordingly, the manuscript should explicitly state that: the direction of association differs depending on the analytical framework, and no definitive conclusion regarding risk reduction or risk increase can be drawn from the current data.

**Response**: We thank the reviewer for highlighting this critical issue. We agree that the direction of association differs between the matched cohort and self-controlled analyses and that these discordant findings preclude a definitive directional conclusion regarding the effect of laryngoplasty on pneumonia risk. In response, we have revised the title to reflect an association rather than a reduction in risk and have modified the Abstract, Discussion, and Conclusions to explicitly state that the direction of association varies depending on the analytical framework applied. We now clearly acknowledge that no definitive conclusion regarding risk reduction or risk increase can be drawn from the present data. We believe these revisions more accurately reflect the methodological limitations of the study and address the reviewer’s concern.

**Changes**:

- The abstract section of the manuscript now contains the following sentence: ‘Given the discordant findings across analytical approaches, no definitive conclusion regarding a preventive effect can be drawn from the present study. Further studies are needed to evaluate long-term outcomes and to clarify causal relationships.’ (page 3, lines 47–49)
- The discussion section of the manuscript now contains the following sentence: ‘In the matched cohort analysis, the incidence of pneumonia after UVFP onset tended to be higher in the treatment group than in the non-treatment group (HR, 1.39; 95% CI, 0.93–2.08; p = 0.098). In contrast, the self-controlled analysis demonstrated a reduction in pneumonia incidence after laryngoplasty compared with the preoperative period within the same individuals (IRR, 0.36; 95% CI: 0.27–0.52; p < 0.001). These findings indicate that the estimated association between laryngoplasty and pneumonia risk is sensitive to the analytical framework applied, and a definitive conclusion regarding risk reduction cannot be drawn from the present data. (page 14-15, lines 268–274)
- The discussion section of the manuscript now contains the following sentence: ‘Third, self-controlled analysis may be susceptible to time-varying confounding and event-dependent exposure. Pneumonia episodes occurring shortly before surgery may have influenced the indication for laryngoplasty and patients’ clinical status may have differed systematically between the pre- and post-surgical periods. These limitations may have biased within-person comparisons. As described previously, the observed reduction in pneumonia incidence after surgery should therefore be interpreted with caution.’ (page 19, lines 364-369)
- The discussion section of the manuscript now contains the following sentence: ‘Notably, the direction of the association differed depending on the analytical framework applied, with discordant findings observed between the matched cohort and self-controlled analyses. Therefore, no definitive conclusion regarding the preventive effect of laryngoplasty on pneumonia can be drawn from the present study. Nevertheless, the use of a large and representative sample provides valuable real-world insights into treatment patterns and pneumonia risk among patients with UVFP. Further studies employing more rigorous causal designs are needed to clarify the true clinical impact of laryngoplasty.’ (page 19-20, lines 374-380)
- The conclusions section of the manuscript now contains the following sentence: ‘This retrospective cohort study, based on a large Japanese insurance claims database, examined the association between laryngoplasty and pneumonia incidence in patients younger than 75 years with UVFP. In the matched cohort analysis, pneumonia incidence tended to be higher in the treatment group, whereas the self-controlled analysis demonstrated a reduction in pneumonia incidence after laryngoplasty within the same individuals. These findings indicate that the observed association depends on the analytical approach applied. Further studies are needed to clarify long-term outcomes and causal relationships.’ (page 20, lines 383-388)
