# Supplementary material for: Association between laryngoplasty and pneumonia incidence in patients with unilateral vocal fold paralysis: A Japanese insurance claims database study
Source: PLoS One. 2026 Jul 2;21(7):e0352874. doi: 10.1371/journal.pone.0352874 (PMC13327127; doi:10.1371/journal.pone.0352874)
Supplement: Peer review history

# Peer review: Decision Letter 0

**DOI:** 10.1371/journal.pone.0352874.r001

**Authors:** Maheshkumar Baladaniya

20 Oct 2025

PONE-D-25-45734

Reduction in Pneumonia Incidence After Laryngoplasty for Unilateral Vocal Fold Paralysis: A Japanese Insurance Claims Database Study

PLOS ONE

Dear Dr. Sano,

Thank you for submitting your manuscript to PLOS ONE. After careful consideration, we feel that it has merit but does not fully meet PLOS ONE’s publication criteria as it currently stands. Therefore, we invite you to submit a revised version of the manuscript that addresses the points raised during the review process.

Please submit your revised manuscript by Dec 04 2025 11:59PM. If you will need more time than this to complete your revisions, please reply to this message or contact the journal office at plosone@plos.org. When you're ready to submit your revision, log on to https://www.editorialmanager.com/pone/ and select the 'Submissions Needing Revision' folder to locate your manuscript file.

Please include the following items when submitting your revised manuscript:

- A rebuttal letter that responds to each point raised by the academic editor and reviewer(s). You should upload this letter as a separate file labeled 'Response to Reviewers'.
- A marked-up copy of your manuscript that highlights changes made to the original version. You should upload this as a separate file labeled 'Revised Manuscript with Track Changes'.
- An unmarked version of your revised paper without tracked changes. You should upload this as a separate file labeled 'Manuscript'.

-->If you would like to make changes to your financial disclosure, please include your updated statement in your cover letter. Guidelines for resubmitting your figure files are available below the reviewer comments at the end of this letter.

If applicable, we recommend that you deposit your laboratory protocols in protocols.io to enhance the reproducibility of your results. Protocols.io assigns your protocol its own identifier (DOI) so that it can be cited independently in the future. For instructions see: https://journals.plos.org/plosone/s/submission-guidelines#loc-laboratory-protocols. Additionally, PLOS ONE offers an option for publishing peer-reviewed Lab Protocol articles, which describe protocols hosted on protocols.io. Read more information on sharing protocols at https://plos.org/protocols?utm_medium=editorial-email&utm_source=authorletters&utm_campaign=protocols.

We look forward to receiving your revised manuscript.

Kind regards,

Maheshkumar Baladaniya

Academic Editor

PLOS ONE

Journal Requirements:

When submitting your revision, we need you to address these additional requirements.

1. Please ensure that your manuscript meets PLOS ONE's style requirements, including those for file naming. The PLOS ONE style templates can be found at

https://journals.plos.org/plosone/s/file?id=wjVg/PLOSOne_formatting_sample_main_body.pdf and

https://journals.plos.org/plosone/s/file?id=ba62/PLOSOne_formatting_sample_title_authors_affiliations.pdf

2. Thank you for stating the following in your Competing Interests section:

“NO authors have competing interests”

Please complete your Competing Interests on the online submission form to state any Competing Interests. If you have no competing interests, please state "The authors have declared that no competing interests exist.", as detailed online in our guide for authors at http://journals.plos.org/plosone/s/submit-now

This information should be included in your cover letter; we will change the online submission form on your behalf.

3. If the reviewer comments include a recommendation to cite specific previously published works, please review and evaluate these publications to determine whether they are relevant and should be cited. There is no requirement to cite these works unless the editor has indicated otherwise.

4. Please review your reference list to ensure that it is complete and correct. If you have cited papers that have been retracted, please include the rationale for doing so in the manuscript text, or remove these references and replace them with relevant current references. Any changes to the reference list should be mentioned in the rebuttal letter that accompanies your revised manuscript. If you need to cite a retracted article, indicate the article’s retracted status in the References list and also include a citation and full reference for the retraction notice.

Additional Editor Comments :

Revision needed as reviewers have suggested points those need to be addressed.

[Note: HTML markup is below. Please do not edit.]

Reviewers' comments:

Reviewer's Responses to Questions

**Comments to the Author**

1. Is the manuscript technically sound, and do the data support the conclusions?

The manuscript must describe a technically sound piece of scientific research with data that supports the conclusions. Experiments must have been conducted rigorously, with appropriate controls, replication, and sample sizes. The conclusions must be drawn appropriately based on the data presented.

Reviewer #1: Yes

Reviewer #2: Yes

**********

2. Has the statistical analysis been performed appropriately and rigorously?

Reviewer #1: Yes

Reviewer #2: Yes

**********

3. Have the authors made all data underlying the findings in their manuscript fully available?

The PLOS Data policy requires authors to make all data underlying the findings described in their manuscript fully available without restriction, with rare exception (please refer to the Data Availability Statement in the manuscript PDF file). The data should be provided as part of the manuscript or its supporting information, or deposited to a public repository. For example, in addition to summary statistics, the data points behind means, medians and variance measures should be available. If there are restrictions on publicly sharing data—e.g. participant privacy or use of data from a third party—those must be specified.

Reviewer #1: Yes

Reviewer #2: Yes

**********

4. Is the manuscript presented in an intelligible fashion and written in standard English?

PLOS ONE does not copyedit accepted manuscripts, so the language in submitted articles must be clear, correct, and unambiguous. Any typographical or grammatical errors should be corrected at revision, so please note any specific errors here.

Reviewer #1: Yes

Reviewer #2: Yes

**********

5. Review Comments to the Author

Please use the space provided to explain your answers to the questions above. You may also include additional comments for the author, including concerns about dual publication, research ethics, or publication ethics. (Please upload your review as an attachment if it exceeds 20,000 characters)

Reviewer #1: MATERIALS AND METHODS:

Kindly explain how treatments were administered for multiple pneumonia incidents. Indicate the comorbidities incorporated in the propensity score model and describe how the covariate balance was visually evaluated by using Love plots or histograms. Even though the threshold for standardized mean difference is suitable, please specify any variables that still showed minor imbalances post-matching. Indicate how absent data were addressed, if relevant. As the immortal time bias will be addressed later, please include a description in the Methods section on how your design took this into account. The pneumonia definition is solid—incorporating a reference that confirms this algorithm in the JMDC database would enhance reliability. Finally, please specify the precise R package utilized for propensity score matching

RESULTS:

Graphs provide valuable information but would improve with larger font sizes for the axis labels and more distinct legends. In addition to IRRs, provide 95% confidence intervals for incidence rates. In the subgroup analysis, specify sample sizes and p-values for the injection laryngoplasty and laryngeal framework surgery groups.

DISCUSSION:

A brief expansion on population and methodological variations that could clarify the differing hazard ratios would improve interpretation. The part regarding clinical implications could further stress whether the noted short-term decrease advocates for earlier surgical procedures or more precise selection of high-risk individuals. The limitations are thorough and straightforward; however, think about positioning the immortal-time-bias explanation earlier in this section to underscore analytic caution. Including a comment on possible supplementary functions of swallowing therapy or post-surgery care would enhance the overall clinical viewpoint.

You might think about referencing the review Heart and Lung Dysfunction Prevention Through Rehabilitation and Physical Therapy Education [ DOI: doi.org/10.47363/JCRRR/2024(5)192] to bolster more general arguments regarding how rehabilitation and the preservation of pulmonary function can enhance respiratory health. Nevertheless, exercise caution not to exaggerate its importance: it addresses general cardiopulmonary rehabilitation, excluding dysphagia or aspiration in vocal fold paralysis. Your main assertions should be supported by more detailed evidence. Utilize this as additional context, rather than a central basis.

Reviewer #2: The topic is clinically important, as aspiration pneumonia remains a key complication in UVFP. The study demonstrates a significant short-term reduction in pneumonia incidence after laryngoplasty, suggesting preventive potential in high-risk groups. However, several clarifications and minor improvements in methodological description and discussion are recommended to strengthen interpretability and transparency.

Comments

It is not entirely clear whether only the first pneumonia event per patient was analyzed or if recurrent events were also included. This detail is critical for interpreting the Poisson regression results.

The manuscript should specify the list of comorbidities used in propensity score calculation (e.g., based on Charlson Comorbidity Index components or ICD-10 codes). This transparency will help readers assess covariate balance validity.

The authors mention possible immortal time bias but only partially address it. Please elaborate on the analytic approach used to minimize this bias, especially given that follow-up starts at different times relative to surgery. Indicate whether a sensitivity analysis excluding early follow-up cases altered the results.

The discussion could better articulate how laryngoplasty reduces pneumonia—beyond glottic closure restoration—by referencing improved airway clearance, cough efficacy, and swallowing biomechanics. This would contextualize the observed reduction in incidence more clearly for clinicians.

The discussion clearly presents the clinical implications of laryngoplasty but lacks integration of prior pathophysiological evidence explaining pulmonary complications following airway obstruction. Inserting the below phrase will strengthen the discussion by linking current findings with previously reported mechanisms of pulmonary complications, thereby providing a coherent physiological basis for the observed reduction in pneumonia incidence.

“Recent study demonstrated how sudden relief of upper airway obstruction can precipitate acute pulmonary compromise due to abrupt shifts in intrathoracic and vascular pressures. These findings underscore the critical importance of maintaining airway stability to prevent secondary respiratory complications [Sheikh et al., 2024, https://doi.org/10.18231/j.ijfcm.2024.028 ]. In line with this pathophysiological understanding, the present study’s observation that laryngoplasty reduces pneumonia incidence further supports the notion that controlled and sustained restoration of airway function plays a preventive role in mitigating pulmonary morbidity.”

Implication

The discussion section misses an explicit linkage between histopathological findings and their clinical implications for patient management. Add below phrase to show practical application to the management of related condition.

“Previous research in poisoning cases identified pulmonary edema, pneumonia, and other respiratory complications as frequent findings [Jain et al., 2024 https://pmc.ncbi.nlm.nih.gov/articles/PMC11513817/ ]. The present study may provide valuable insights for clinicians managing poisoning cases, as early recognition and prevention of pulmonary complications such as pneumonia could help reduce morbidity and mortality in similar clinical settings.”

**********

6. PLOS authors have the option to publish the peer review history of their article (what does this mean?). If published, this will include your full peer review and any attached files.

If you choose “no”, your identity will remain anonymous but your review may still be made public.

**Do you want your identity to be public for this peer review?**  For information about this choice, including consent withdrawal, please see our Privacy Policy.

Reviewer #1: No

Reviewer #2: No

**********

[NOTE: If reviewer comments were submitted as an attachment file, they will be attached to this email and accessible via the submission site. Please log into your account, locate the manuscript record, and check for the action link "View Attachments". If this link does not appear, there are no attachment files.]

To ensure your figures meet our technical requirements, please review our figure guidelines: https://journals.plos.org/plosone/s/figures

You may also use PLOS’s free figure tool, NAAS, to help you prepare publication quality figures: https://journals.plos.org/plosone/s/figures#loc-tools-for-figure-preparation.

NAAS will assess whether your figures meet our technical requirements by comparing each figure against our figure specifications.

Attachment Submitted filename: Comments.docx

# Author comment: Author response to Decision Letter 1

**DOI:** 10.1371/journal.pone.0352874.r002

18 Nov 2025

Thank you very much for taking the time to review our work. Your comments are appreciated and addressed below;

Note: the quotes from reviewers are in normal font, and our response is in navy blue font. The changes are highlighted in the revised manuscript.

Reviewer #1:

1. MATERIALS AND METHODS:

Kindly explain how treatments were administered for multiple pneumonia incidents. Indicate the comorbidities incorporated in the propensity score model and describe how the covariate balance was visually evaluated by using Love plots or histograms. Even though the threshold for standardized mean difference is suitable, please specify any variables that still showed minor imbalances post-matching. Indicate how absent data were addressed, if relevant. As the immortal time bias will be addressed later, please include a description in the Methods section on how your design took this into account. The pneumonia definition is solid—incorporating a reference that confirms this algorithm in the JMDC database would enhance reliability. Finally, please specify the precise R package utilized for propensity score matching.

Response: First, we really appreciate you taking the time to offer us your comments. We thank the reviewer for thoughtful comments about our work. We have incorporated changes that reflect the detailed suggestions you have graciously provided. We also hope that our edits and the responses we provided below satisfactorily address all the issues and concerns you and the reviewers have noted.

Changes:

• We now provide a list of the antibiotics used to treat multiple pneumonia incidents as the supplementary material (S1 Table) and cited in the main text (page 6, lines 119).

• All comorbidities included in the propensity score model are now specified in the manuscript (page 7, lines 154–157). We have also added a Love plot to visually evaluate covariate balance, as shown in S1 Fig. The result section of the manuscript now contains the following sentence: ‘some variables, such as stroke and dysphagia, still exceeded the 0.1 threshold (supplementary material, S1 Fig)’ (page 9, line 175-176).

• As the present study included only complete data, no imputation for missing values was required.

• To address the immortal time bias caused by differential censoring, a sensitivity analysis was conducted that excluded cases in which the post-treatment period was shorter than the pre-treatment period. The materials and methods section of the manuscript now contains the following sentence: ‘To address potential immortal time bias related to differential censoring or loss to follow-up, a sensitivity analysis was conducted by excluding cases in which the post-treatment period was shorter than the pre-treatment period’ (page 6, lines 121–124).

• The pneumonia definition algorithm used in this study has been validated in previous studies utilizing the JMDC database. The materials and methods section of the manuscript now contains the following sentence: ‘This algorithm in the JMDC database has been used in previous studies21,24’ (page 6, line 121).

• We now specify the R package in the Statistical Analysis section (page 7, line 137) as you suggested.

2. RESULTS:

Graphs provide valuable information but would improve with larger font sizes for the axis labels and more distinct legends. In addition to IRRs, provide 95% confidence intervals for incidence rates. In the subgroup analysis, specify sample sizes and p-values for the injection laryngoplasty and laryngeal framework surgery groups.

Response: We appreciate the reviewer’s constructive comment. We revised the figures to improve readability and clarity.

Changes:

• The font sizes of the axis labels and legends in the Kaplan–Meier plots were increased for better readability (Fig 3, S3 Fig).

• 95% confidence intervals for incidence rates were provided (Fig 4, S2 Fig, S2 Table).

• In the subgroup analysis, sample size and p-values for the injection laryngoplasty and laryngeal framework surgery groups were added. The result section of the manuscript now contains the following sentence: ‘In the subgroup analysis by surgical procedure, both IL (n= 104) and LFS (n=104) were associated with a reduction in pneumonia incidence after treatment (S2 Table). The IRR decreased from 0.34 to 0.16 for IL (IRR, 0.47; 95% CI, 0.28–0.79; p = 0.003) and from 0.22 to 0.12 for LFS (IRR, 0.54; 95% CI, 0.31–0.97; p = 0.031)’. (page 12, line 218-221).

3. DISUCUSSION :

A brief expansion on population and methodological variations that could clarify the differing hazard ratios would improve interpretation. The part regarding clinical implications could further stress whether the noted short-term decrease advocates for earlier surgical procedures or more precise selection of high-risk individuals. The limitations are thorough and straightforward; however, think about positioning the immortal-time-bias explanation earlier in this section to underscore analytic caution. Including a comment on possible supplementary functions of swallowing therapy or post-surgery care would enhance the overall clinical viewpoint.

You might think about referencing the review Heart and Lung Dysfunction Prevention Through Rehabilitation and Physical Therapy Education [ DOI: doi.org/10.47363/JCRRR/2024(5)192] to bolster more general arguments regarding how rehabilitation and the preservation of pulmonary function can enhance respiratory health. Nevertheless, exercise caution not to exaggerate its importance: it addresses general cardiopulmonary rehabilitation, excluding dysphagia or aspiration in vocal fold paralysis. Your main assertions should be supported by more detailed evidence. Utilize this as additional context, rather than a central basis.

Response: We appreciate this valuable suggestion. We revised the discussion section to clarify population and methodological differences, highlight the clinical implications of early surgical intervention, and expand the discussion on postoperative management and rehabilitation.

Changes:

• The discussion was expanded to address both population and methodological variations in order to clarify the differences in hazard ratios compared with previous reports. The discussion section of the manuscript now contains the following sentence: ‘The discrepancy between these findings may result from differences in patient populations. Particularly, the present study included patients with more severe cases who were at higher risk of developing pneumonia reflecting real-world clinical practice, as several Japanese studies have reported the therapeutic efficacy of laryngoplasty in UVFP patients with an aspiration risk27-29. In addition, variations in the definition of pneumonia and in the classification of surgical procedures may have led to differences in event ascertainment’ (page 14, lines 253–258).

• The clinical implication that early surgical intervention should be appropriately performed in high-risk patients was emphasized. The discussion section of the manuscript now contains the following sentence: ‘These results suggest that performing IL or LFS within approximately six months of onset may help reduce pneumonia incidence among high-risk individuals with UVFP, such as older patients and those with dysphagia or cancer, as identified in the Poisson regression analysis. Our findings emphasize the importance of appropriate patient selection and early surgical intervention for UVFP to prevent pneumonia in real-world clinical settings’ (page 18, lines 332-336).

• The rationale and interpretation of the sensitivity analysis were repositioned earlier in the discussion to improve clarity regarding immortal time bias. The discussion section of the manuscript now contains the following sentence: ‘However, censoring events such as death or disenrollment from employees’ health insurance could not be distinguished in this database. Because censoring does not occur before laryngoplasty, a decrease in pneumonia incidence observed after surgery could be influenced by immortal time bias. To address this potential bias, we performed a sensitivity analysis that excluded cases in which the post-treatment period was shorter than the pre-treatment period. The results remained consistent, indicating that the observed decrease in pneumonia incidence after surgery was robust’ (page 15, lines 268–273).

• A comment on the potential influence and supplementary functions of swallowing therapy and post-surgical care was added. The discussion section of the manuscript now contains the following sentence: ‘Furthermore, swallowing therapy, such as chin tuck, neck extension, head turn, supraglottic swallow, super-supraglottic swallow, and dietary modification has been shown to be effective for UVFP with dysphagia1,43 ‘ (page 17, line 307-309) ’However, the effect of injected hyaluronic acid filler is generally reported to last for only 4–6 months, after which natural absorption may lead to recurrence of aspiration49. Therefore, it is desirable to conduct objective evaluations such as fiberoptic endoscopic evaluation of swallowing (FEES) or video fluoroscopic swallow study (VFSS) before and after surgery to assess swallowing function and the presence of aspiration over time50’ (page 17-18, lines 322–326).

• Relevant literature on physical therapy approaches aimed at preserving pulmonary function was referenced. The discussion section of the manuscript now contains the following sentence: ‘In addition, pulmonary rehabilitation techniques such as straw breathing, pursued-lip breathing, and equal breathing enhance diaphragmatic activity and increase lung capacity, thereby contributing to the preservation of pulmonary function44. Due to the characteristics of the database in the present study, we were unable to identify the underlying causes of non-iatrogenic paralysis or determine whether patients received any rehabilitation. Therefore, to isolate the effect of laryngoplasty and minimize confounding factors from other surgical swallowing interventions, we excluded patients who underwent surgeries to improve swallowing. Appropriate postoperative care following laryngoplasty may have also contributed to the observed reduction in pneumonia incidence’ (page 17, lines 309–316).

Reviewer #2:

The topic is clinically important, as aspiration pneumonia remains a key complication in UVFP. The study demonstrates a significant short-term reduction in pneumonia incidence after laryngoplasty, suggesting preventive potential in high-risk groups. However, several clarifications and minor improvements in methodological description and discussion are recommended to strengthen interpretability and transparency.

Response: We really appreciate you taking the time to offer us your comments. We thank the reviewer for thoughtful comments about our work. We have incorporated changes that reflect the detailed suggestions you have graciously provided. We also hope that our edits and the responses we provided below satisfactorily address all the issues and concerns you and the reviewers have noted.

1. It is not entirely clear whether only the first pneumonia event per patient was analyzed or if recurrent events were also included. This detail is critical for interpreting the Poisson regression results.

Response: We appreciate this valuable comment. We specified that recurrent pneumonia episodes during the follow-up period were included in the analysis as count data to ensure accurate interpretation of the Poisson regression results.

Changes: The methods section of the manuscript now contains the following sentence: ‘Each pneumonia episode accompanied by antibiotic prescription was considered an independent recurrent event during the follow-up period, and the number of such events was analyzed as count data’ (page 6, lines 119–121).

2. The manuscript should specify the list of comorbidities used in propensity score calculation (e.g., based on Charlson Comorbidity Index components or ICD-10 codes). This transparency will help readers assess covariate balance validity.

Response: We appreciate this important comment. To improve transparency and reproducibility, we specified the list of comorbidities and their corresponding ICD-10 codes used in the propensity score model , which are now provided in the supplementary material (S1 Table).

Changes: The list of ICD-10 codes used to define comorbidities has been added. The materials and methods section of the manuscript now contains the following sentence: ‘Comorbidities were also identified according to ICD-10 codes listed in the supplementary material (S1 Table)’ (page 7, lines 136–137).

3. The authors mention possible immortal time bias but only partially address it. Please elaborate on the analytic approach used to minimize this bias, especially given that follow-up starts at different times relative to surgery. Indicate whether a sensitivity analysis excluding early follow-up cases altered the results

Response: We appreciate this crucial point. To address this issue, we elaborated on the analytic approach used to minimize immortal time bias and clarified the results of the corresponding sensitivity analysis. Specifically, we conducted a sensitivity analysis that excluded cases with short post-treatment follow-up periods to reduce the potential effect of immortal time bias and confirmed that the findings from this sensitivity analysis were consistent with those of the primary outcome, as shown at the end of the results section and in the supplementary material (S2 Fig).

Changes:

• The rationale and description of the sensitivity analysis to minimize immortal time bias was added. The materials and methods section of the manuscript now contains the following sentence: ‘To address potential immortal time bias related to differential censoring or loss to follow-up, a sensitivity analysis was conducted by excluding cases in which the post-treatment period was shorter than the pre-treatment period’ (page 6, lines 121–124). The results section of the manuscript now contains the following sentence: ‘Of the 230 patients in the treatment group, 53 were excluded due to a post-treatment period shorter than the pre-treatment period. Among the remaining 177 patients, the IRR of pneumonia after surgery was significantly lower than that before surgery (0.54; 95% CI: 0.31–0.97; p < 0.001), indicating a consistent reduction in pneumonia incidence following laryngoplasty (supplementary material, S2 Fig)’ (page 13, lines 234–238).

• The interpretation of the analysis and the robustness of the results were described. The discussion section of the manuscript now contains the following sentence: ‘Because censoring does not occur before laryngoplasty, a decrease in pneumonia incidence observed after surgery could be influenced by immortal time bias. To address this potential bias, we performed a sensitivity analysis that excluded cases in which the post-treatment period was shorter than the pre-treatment period. The results remained consistent, indicating that the observed decrease in pneumonia incidence after surgery was robust’ (page 15, lines 269–273).

4. The discussion could better articulate how laryngoplasty reduces pneumonia—beyond glottic closure restoration—by referencing improved airway clearance, cough efficacy, and swallowing biomechanics. This would contextualize the observed reduction in incidence more clearly for clinicians.

Response: We appreciate this insightful comment. To clarify the mechanisms through which laryngoplasty may reduce pneumonia, we expanded the discussion and incorporated relevant evidence. We referenced studies demonstrating that laryngoplasty enhances airway clearance and cough strength by improving subglottic pressure and glottic function. We additionally cited literature investigating improvements in swallowing function following laryngoplasty, providing further support for the observed reduction in pneumonia incidence.

Changes:

• A more detailed explanation by referencing evidence demonstrating that laryngoplasty improves airway clearance and cough efficacy was provided. The discussion section of the manuscript now contains the following sentence: ‘Watanabe et al. reported that the combination of arytenoid adduction and medialization laryngoplasty significantly improved coug

Attachment Submitted filename: Author responce to reviewers 20251111.docx

# Peer review: Decision Letter 1

**DOI:** 10.1371/journal.pone.0352874.r003

**Authors:** Maheshkumar Baladaniya, Maheshkumar Baladaniya

28 Dec 2025

<div>PONE-D-25-45734R1

Reduction in Pneumonia Incidence After Laryngoplasty for Unilateral Vocal Fold Paralysis: A Japanese Insurance Claims Database Study

PLOS One

Dear Dr. Sano,

Thank you for submitting your manuscript to PLOS ONE. After careful consideration, we feel that it has merit but does not fully meet PLOS ONE’s publication criteria as it currently stands. Therefore, we invite you to submit a revised version of the manuscript that addresses the points raised during the review process.

Please submit your revised manuscript by Feb 11 2026 11:59PM. If you will need more time than this to complete your revisions, please reply to this message or contact the journal office at plosone@plos.org. When you're ready to submit your revision, log on to https://www.editorialmanager.com/pone/ and select the 'Submissions Needing Revision' folder to locate your manuscript file.

Please include the following items when submitting your revised manuscript:

- A letter that responds to each point raised by the academic editor and reviewer(s). You should upload this letter as a separate file labeled 'Response to Reviewers'.
- A marked-up copy of your manuscript that highlights changes made to the original version. You should upload this as a separate file labeled 'Revised Manuscript with Track Changes'.
- An unmarked version of your revised paper without tracked changes. You should upload this as a separate file labeled 'Manuscript'.

-->If you would like to make changes to your financial disclosure, please include your updated statement in your cover letter. Guidelines for resubmitting your figure files are available below the reviewer comments at the end of this letter.

If applicable, we recommend that you deposit your laboratory protocols in protocols.io to enhance the reproducibility of your results. Protocols.io assigns your protocol its own identifier (DOI) so that it can be cited independently in the future. For instructions see: https://journals.plos.org/plosone/s/submission-guidelines#loc-laboratory-protocols. Additionally, PLOS ONE offers an option for publishing peer-reviewed Lab Protocol articles, which describe protocols hosted on protocols.io. Read more information on sharing protocols at https://plos.org/protocols?utm_medium=editorial-email&utm_source=authorletters&utm_campaign=protocols.

We look forward to receiving your revised manuscript.

Kind regards,

Maheshkumar Baladaniya

Academic Editor

PLOS One

Journal Requirements:

If the reviewer comments include a recommendation to cite specific previously published works, please review and evaluate these publications to determine whether they are relevant and should be cited. There is no requirement to cite these works unless the editor has indicated otherwise.

Please review your reference list to ensure that it is complete and correct. If you have cited papers that have been retracted, please include the rationale for doing so in the manuscript text, or remove these references and replace them with relevant current references. Any changes to the reference list should be mentioned in the rebuttal letter that accompanies your revised manuscript. If you need to cite a retracted article, indicate the article’s retracted status in the References list and also include a citation and full reference for the retraction notice.

Additional Editor Comments:

There are some issues which are critical to publish the paper. Please address those issues.

[Note: HTML markup is below. Please do not edit.]

Reviewers' comments:

Reviewer's Responses to Questions

**Comments to the Author**

1. If the authors have adequately addressed your comments raised in a previous round of review and you feel that this manuscript is now acceptable for publication, you may indicate that here to bypass the “Comments to the Author” section, enter your conflict of interest statement in the “Confidential to Editor” section, and submit your "Accept" recommendation.

Reviewer #3: All comments have been addressed

Reviewer #4: (No Response)

**********

2. Is the manuscript technically sound, and do the data support the conclusions?

The manuscript must describe a technically sound piece of scientific research with data that supports the conclusions. Experiments must have been conducted rigorously, with appropriate controls, replication, and sample sizes. The conclusions must be drawn appropriately based on the data presented.

Reviewer #3: Yes

Reviewer #4: Yes

**********

3. Has the statistical analysis been performed appropriately and rigorously?

Reviewer #3: Yes

Reviewer #4: No

**********

4. Have the authors made all data underlying the findings in their manuscript fully available?

The PLOS Data policy requires authors to make all data underlying the findings described in their manuscript fully available without restriction, with rare exception (please refer to the Data Availability Statement in the manuscript PDF file). The data should be provided as part of the manuscript or its supporting information, or deposited to a public repository. For example, in addition to summary statistics, the data points behind means, medians and variance measures should be available. If there are restrictions on publicly sharing data—e.g. participant privacy or use of data from a third party—those must be specified.

Reviewer #3: Yes

Reviewer #4: No

**********

5. Is the manuscript presented in an intelligible fashion and written in standard English?

PLOS ONE does not copyedit accepted manuscripts, so the language in submitted articles must be clear, correct, and unambiguous. Any typographical or grammatical errors should be corrected at revision, so please note any specific errors here.

Reviewer #3: Yes

Reviewer #4: Yes

**********

6. Review Comments to the Author

Please use the space provided to explain your answers to the questions above. You may also include additional comments for the author, including concerns about dual publication, research ethics, or publication ethics. (Please upload your review as an attachment if it exceeds 20,000 characters)

Reviewer #3: I was not involved in the first round of peer review and have joined the review process from this revision. Therefore, I focused my evaluation on whether the issues raised in the previous round have been appropriately addressed in the current revision. From this perspective, I find that the authors have thoroughly considered the previous reviewer’s comments and have incorporated the requested revisions appropriately.

On the other hand, the Discussion section has become quite long and redundant, which I personally find a bit concerning. However, this appears to be a result of the authors’ sincere efforts to address the prior comments. Whether further condensation of the Discussion is necessary may be best left to the editor’s discretion.

Reviewer #4: Review comments

Thank you for the opportunity to review this revised manuscript. The topic is clinically meaningful; however, there remain important methodological and interpretational issues that warrant clarification before the manuscript can be considered for publication. My major comments are as follows.

Major

1. The study presents three different analytical approaches:

(1) a propensity score–matched cohort analysis with HRs,

(2) a pre–post self-controlled IRR analysis among treated patients, and

(3) a Poisson regression evaluating baseline risk factors for pneumonia (Table 3).

However, the manuscript does not clearly articulate the hierarchical role of these analyses or define the primary estimand. These analytical components answer different questions (between-person vs within-person comparisons), and their relationship should be clarified. I strongly recommend explicitly designating the primary analysis and explaining how the other analyses support the main interpretation.

2. S1 Fig shows standardized mean differences before and after matching, revealing that several important covariates (e.g., age category, COPD, esophageal cancer) remain above the usual 0.1 threshold even after matching. In addition, dysphagia, one of the strongest known risk factors for pneumonia, is not appropriately adjusted for in the matched cohort analysis and does not appear to be fully balanced after matching. Given its major clinical and statistical impact on pneumonia risk, failure to adjust for dysphagia meaningfully limits interpretability of the treatment effect estimates. This point should be addressed directly, and additional adjustment or sensitivity analyses should be considered.

The authors would be better to:

• acknowledge residual imbalance and possible residual confounding,

• justify whether additional adjustment was considered, and

• summarize the final covariate balance in the main text.

This is especially important because the matched cohort HR is one of the key analyses.

3. Table 3 evaluates baseline predictors of pneumonia incidence using Poisson regression, but the aim and analytic role of this model are not well explained. The analysis is not an adjustment of the treatment effect but rather a cross-sectional incidence predictor model.

To avoid reader confusion, I recommend:

• clearly stating the purpose of Table 3, or

• moving Table 3 to the Supplementary materials if it is not central to the study’s conclusions.

4. Fig4 and S2 Fig shows a reduced IRR after surgery, but this finding is highly susceptible to confounding by indication and event-dependent exposure. Pneumonia occurring shortly before laryngoplasty often represents the clinical deterioration that motivates surgery, inflating the preoperative incidence rate and biasing the IRR downward.

I recommend a sensitivity analysis that:

• excludes pneumonia events (and person-time) occurring in the a couple of months immediately prior to surgery, or

• treats this period as a separate "high-risk" window.

This is consistent with practices in self-controlled designs where event-dependent exposure is a risk.

The manuscript should also incorporate an expanded discussion of time-varying confounding and reverse causation.

5. The manuscript should address the known limitations of using claims-based pneumonia diagnoses in Japanese databases (JMDC). Although DPC-based inpatient pneumonia codes (J12–J18) have shown reasonably high PPV (approximately 70–90%) in validation studies, the accuracy of pneumonia coding in broader claims datasets such as JMDC or NDB has not been systematically validated. Aspiration pneumonia (J69) in particular is known to have lower PPV (around 60–75%).

Given these limitations, the authors should explicitly acknowledge the possibility of outcome misclassification, especially because pneumonia diagnosis is the primary endpoint.

6. Lack of adjustment for preoperative pneumonia severity

The manuscript reports the percentage of patients with pneumonia before surgery but does not address the severity of preoperative pneumonia, including: pneumonia frequency (single vs recurrent episodes), oxygen requirement, hospitalization status, use of systemic antibiotics, laboratory or imaging indicators of severity.

These factors may strongly influence both the likelihood of receiving surgery and the postoperative pneumonia risk, yet they were not included in the matching algorithm or adjusted for in the analyses.

As a result, unmeasured confounding related to preoperative disease severity is likely, and the authors should discuss this limitation and consider whether additional adjustment or stratified analyses are feasible.

7. The manuscript would benefit from a strengthened Limitations section, including:

• residual confounding due to imperfect PS matching,

• susceptibility of pre–post IRR to time-varying confounding and confounding by indication,

• risk of event-dependent exposure, and

• outcome misclassification inherent to claims data.

Minor

Fig 1 and 2 are not available in the manuscript.

**********

7. PLOS authors have the option to publish the peer review history of their article (what does this mean?). If published, this will include your full peer review and any attached files.

If you choose “no”, your identity will remain anonymous but your review may still be made public.

**Do you want your identity to be public for this peer review?**  For information about this choice, including consent withdrawal, please see our Privacy Policy.

Reviewer #3: **Yes:**  Yoshiki Kusama

Reviewer #4: No

**********

[NOTE: If reviewer comments were submitted as an attachment file, they will be attached to this email and accessible via the submission site. Please log into your account, locate the manuscript record, and check for the action link "View Attachments". If this link does not appear, there are no attachment files.]

To ensure your figures meet our technical requirements, please review our figure guidelines: https://journals.plos.org/plosone/s/figures

You may also use PLOS’s free figure tool, NAAS, to help you prepare publication quality figures: https://journals.plos.org/plosone/s/figures#loc-tools-for-figure-preparation.

NAAS will assess whether your figures meet our technical requirements by comparing each figure against our figure specifications.

Attachment Submitted filename: Review comments.docx

# Author comment: Author response to Decision Letter 2

**DOI:** 10.1371/journal.pone.0352874.r004

13 Jan 2026

Thank you very much for taking the time to review our work. Your comments are appreciated and addressed below;

Note: the quotes from reviewers are in normal font, and our response is in navy blue font. The changes are highlighted in the revised manuscript.

Reviewer #3:

I was not involved in the first round of peer review and have joined the review process from this revision. Therefore, I focused my evaluation on whether the issues raised in the previous round have been appropriately addressed in the current revision. From this perspective, I find that the authors have thoroughly considered the previous reviewer’s comments and have incorporated the requested revisions appropriately.

On the other hand, the Discussion section has become quite long and redundant, which I personally find a bit concerning. However, this appears to be a result of the authors’ sincere efforts to address the prior comments. Whether further condensation of the Discussion is necessary may be best left to the editor’s discretion.

Response:

We thank the reviewer for the careful evaluation of the revised manuscript and for the positive assessment that the concerns raised in the previous round were appropriately addressed.

We also appreciate the comment regarding the length and potential redundancy of the Discussion section. In the revised version, we have carefully reviewed the Discussion and condensed overlapping statements where possible. Please note that we included additional discussion in the revised manuscript, as requested in the current review.

Reviewer #4:

Thank you for the opportunity to review this revised manuscript. The topic is clinically meaningful; however, there remain important methodological and interpretational issues that warrant clarification before the manuscript can be considered for publication. My major comments are as follows.

Response: We really appreciate you taking the time to offer us your comments. We thank the reviewer for thoughtful comments about our work. We have incorporated changes that reflect the detailed suggestions you have graciously provided. We also hope that our edits and the responses we provided below satisfactorily address all the issues and concerns you and the reviewers have noted.

Major

1. The study presents three different analytical approaches:

(1) a propensity score–matched cohort analysis with HRs,

(2) a pre–post self-controlled IRR analysis among treated patients, and

(3) a Poisson regression evaluating baseline risk factors for pneumonia (Table 3).

However, the manuscript does not clearly articulate the hierarchical role of these analyses or define the primary estimand. These analytical components answer different questions (between-person vs within-person comparisons), and their relationship should be clarified. I strongly recommend explicitly designating the primary analysis and explaining how the other analyses support the main interpretation.

Response: We appreciate this important comment. We agree that the initial version of the manuscript did not sufficiently clarify the hierarchical role of the different analytical approaches or explicitly define the primary estimand.

To address this concern, we have revised the materials and methods section to clearly designate the pre–post self-controlled incidence rate ratio (IRR) analysis among treated patients as the primary analysis. This analysis estimates within-person changes in pneumonia incidence before and after laryngoplasty and was designed to provide a causal estimate of the treatment effect while minimizing confounding by time-invariant individual characteristics.

The propensity score–matched cohort analysis comparing the cumulative incidence of pneumonia between the treatment and non-treatment groups is now explicitly described as a secondary analysis, intended to complement the primary analysis by assessing potential differences in pneumonia risk at the population level.

In addition, the Poisson regression model evaluating baseline predictors of pneumonia has been repositioned as an exploratory analysis, aimed at identifying baseline risk factors and vulnerable subpopulations among patients with UVFP, rather than serving as an adjustment model for treatment effect estimation.

These revisions clarify the distinct objectives of each analytical approach and their respective roles in supporting the overall interpretation of the study findings.

Changes: The materials and methods section of the manuscript now contains the following sentence:

(1) ‘As the primary analysis, we conducted a self-controlled analysis within the treatment group to compare the incidence of pneumonia before and after laryngoplasty. This analysis aimed to evaluate within-person changes in pneumonia incidence associated with the period before and after surgery, thereby minimizing confounding by time-invariant individual characteristics.’ (page 6, lines 119–122).

(2) ‘As the secondary analysis, we compared the cumulative incidence of pneumonia between the treatment group and the non-treatment group, with follow-up starting from a predefined index date, defined as the onset of UVFP. This analysis aimed to assess potential differences in pneumonia risk at the population level between patients who underwent laryngoplasty and those who did not’ (page 6-7, lines 126–130).

(3) ‘In addition, as an exploratory analysis, we examined baseline predictors of pneumonia in the overall cohort. This analysis aimed to identify vulnerable subpopulations at higher risk of pneumonia among patients with UVFP and to inform appropriate patient selection for future preventive interventions’ (page 7, lines 130–133).

2. S1 Fig shows standardized mean differences before and after matching, revealing that several important covariates (e.g., age category, COPD, esophageal cancer) remain above the usual 0.1 threshold even after matching. In addition, dysphagia, one of the strongest known risk factors for pneumonia, is not appropriately adjusted for in the matched cohort analysis and does not appear to be fully balanced after matching. Given its major clinical and statistical impact on pneumonia risk, failure to adjust for dysphagia meaningfully limits interpretability of the treatment effect estimates. This point should be addressed directly, and additional adjustment or sensitivity analyses should be considered.

The authors would be better to:

acknowledge residual imbalance and possible residual confounding,

justify whether additional adjustment was considered, and

summarize the final covariate balance in the main text.

This is especially important because the matched cohort HR is one of the key analyses.

Response: We thank the reviewer for this valuable comment. We agree that several important covariates associated with pneumonia risk were not fully balanced after propensity score matching and that additional adjustment for these variables was warranted.

To address this concern, we conducted a sensitivity analysis using multivariable Cox proportional hazards models that additionally included the covariates with standardized mean differences greater than 0.1 after matching, specifically stroke and dysphagia. In the results section, we now explicitly acknowledge the presence of residual imbalance and potential residual confounding and report the adjusted hazard ratios derived from this analysis. Importantly, the adjusted hazard ratio for pneumonia was similar in direction and magnitude to that observed in the propensity score–matched secondary analysis, supporting the interpretability of the between-group comparison of pneumonia incidence.

Changes:

(1) The materials and methods section of the manuscript now contains the following sentence: ‘Furthermore, if any covariates remained insufficiently balanced after PS matching, we performed additional adjustment using multivariable Cox proportional hazards models including these covariates, as recommended in previous studies to mitigate residual confounding in matched observational studies[27, 28]’ (page 8, lines 163–166).

(2) The results section of the manuscript now contains the following sentence: ‘Furthermore, in multivariable Cox proportional hazards models that additionally adjusted for stroke and dysphagia, which remained imbalanced after PS matching (SMD >0.1), the treatment group tended to have a higher incidence of pneumonia compared with the non-treatment group; however, this difference was not statistically significant (adjusted HR, 1.35; 95% CI, 0.90–2.01; p = 0.149) (S4 Table)’ (page 14, lines 265–269).

(3) The discussion section of the manuscript now contains the following sentence: ‘As a sensitivity analysis, we applied a double-adjustment approach using multivariable Cox proportional hazards models that additionally included these covariates. Although the direction of the hazard ratio for pneumonia was consistent with the results of the secondary analysis, residual confounding cannot be fully excluded’ (page 20, lines 390–393).

3. Table 3 evaluates baseline predictors of pneumonia incidence using Poisson regression, but the aim and analytic role of this model are not well explained. The analysis is not an adjustment of the treatment effect but rather a cross-sectional incidence predictor model. To avoid reader confusion, I recommend:

・clearly stating the purpose of Table 3, or

・moving Table 3 to the Supplementary materials if it is not central to the study’s conclusions.

Response: We appreciate this helpful comment. We agree that the Poisson regression analysis evaluating baseline predictors of pneumonia was not intended to estimate the treatment effect and may have caused confusion if presented alongside the primary and secondary analyses. To clarify its analytic role, we have repositioned this analysis as an exploratory analysis and moved the corresponding table to the Supplementary Materials. This analysis is now presented solely to describe baseline risk factors for pneumonia among patients with UVFP, rather than as an adjustment or supportive analysis of the treatment effect.

Changes:

・The Poisson regression analysis of baseline predictors of pneumonia has been moved from the main text to the Supplementary Materials (S3 Table). (page 13, line 247).

4. Fig4 and S2 Fig shows a reduced IRR after surgery, but this finding is highly susceptible to confounding by indication and event-dependent exposure. Pneumonia occurring shortly before laryngoplasty often represents the clinical deterioration that motivates surgery, inflating the preoperative incidence rate and biasing the IRR downward. I recommend a sensitivity analysis that:

・excludes pneumonia events (and person-time) occurring in the a couple of months immediately prior to surgery, or

・treats this period as a separate "high-risk" window.

This is consistent with practices in self-controlled designs where event-dependent exposure is a risk. The manuscript should also incorporate an expanded discussion of time-varying confounding and reverse causation.

Response: We appreciate this insightful comment. We agree that the observed reduction in the incidence rate ratio (IRR) in the pre–post comparison may be susceptible to event-dependent exposure, particularly if pneumonia events occurring shortly before surgery influenced the clinical decision to perform laryngoplasty. Such a mechanism could bias the preoperative pneumonia incidence upward and lead to an overestimation of the apparent treatment effect.

To address this concern, we conducted an additional sensitivity analysis in which pneumonia events occurring within the 2 months immediately preceding laryngoplasty, as well as the corresponding person-time during this period, were excluded as the reviewer suggested. In addition, we expanded the discussion section to explicitly consider the possibility that the observed reduction in IRR in the primary analysis may have been influenced by time-varying confounding and reverse causation.

Importantly, even under this more conservative assumption, the incidence of pneumonia after laryngoplasty remained lower than that before surgery, demonstrating a consistent direction of association. We believe that these additional analyses and the expanded discussion strengthen the robustness and interpretability of the self-controlled analysis.

Changes:

The material and methods section of the manuscript now contains the following sentence: ‘In the primary analysis, the self-controlled comparison of pneumonia incidence before and after laryngoplasty may be susceptible to confounding if pneumonia events occurring shortly before surgery influence the indication for laryngoplasty. To address this concern, we conducted a sensitivity analysis in which pneumonia events occurring within the 2 months immediately preceding laryngoplasty, as well as the corresponding person-time during this period, were excluded from the analysis’ (page 8, lines 156–160).

(1) The results section of the manuscript now contains the following sentence: ‘Of the 230 patients in the treatment group, 67 were excluded from the analysis because their preoperative observation period was shorter than 2 months. Among the remaining 163 patients, comparison of the period from the index date to 2 months before surgery with the postoperative observation period showed that the incidence of pneumonia was significantly lower after laryngoplasty than before surgery (IRR 0.55; 95% CI, 0.37–0.83; p = 0.004) (S3 Fig)’ (page 14, lines257–261).

(2) The discussion section of the manuscript now contains the following sentence: ‘Although the self-controlled analysis in this study minimizes time-invariant confounding through within-person comparisons, it may remain susceptible to time-varying confounding and reverse causation. In particular, pneumonia episodes occurring shortly before laryngoplasty may influence the decision to perform surgery, resulting in event-dependent exposure and a potential overestimation of preoperative pneumonia incidence. To address this concern, we conducted a sensitivity analysis excluding pneumonia events occurring within the 2 months immediately preceding laryngoplasty, as well as the corresponding person-time. In this analysis, the incidence of pneumonia after surgery remained lower than that before surgery; however, the magnitude of the reduction was attenuated, with the IRR increasing from 0.36 in the primary analysis to 0.55. This result suggests that treatment effect estimates using the time of surgery as the reference point may be partially influenced by time-varying confounding related to perioperative events’ (page 16, lines 297–306).

(3) The discussion section of the manuscript now contains the following sentence: ‘Taken together, these sensitivity analyses conducted under different assumptions consistently showed a lower incidence of pneumonia after surgery, suggesting that the primary findings of this study are robust and not driven by specific time-related biases, including time-varying confounding or immortal time bias’ (page 16, lines 311–314).

5. The manuscript should address the known limitations of using claims-based pneumonia diagnoses in Japanese databases (JMDC). Although DPC-based inpatient pneumonia codes (J12–J18) have shown reasonably high PPV (approximately 70–90%) in validation studies, the accuracy of pneumonia coding in broader claims datasets such as JMDC or NDB has not been systematically validated. Aspiration pneumonia (J69) in particular is known to have lower PPV (around 60–75%).

Given these limitations, the authors should explicitly acknowledge the possibility of outcome misclassification, especially because pneumonia diagnosis is the primary endpoint.

Response: The reviewer has raised an important point here. We agree that, at present, the diagnostic accuracy of pneumonia coding in large claims databases such as JMDC or the National Database has not been fully validated. In this study, pneumonia outcomes were identified using an algorithm that combined diagnostic codes with records of systemic antibiotic prescriptions; however, we acknowledge that outcome misclassification may still have occurred despite this approach. In response to this concern, we have strengthened the Limitations section to explicitly acknowledge the potential for mis

Attachment Submitted filename: Author responce to reviewers 20260113ver2.docx

# Peer review: Decision Letter 2

**DOI:** 10.1371/journal.pone.0352874.r005

**Authors:** Maheshkumar Baladaniya, Maheshkumar Baladaniya, Maheshkumar Baladaniya

2 Mar 2026

PONE-D-25-45734R2

Reduction in Pneumonia Incidence After Laryngoplasty for Unilateral Vocal Fold Paralysis: A Japanese Insurance Claims Database Study

PLOS One

Dear Dr. Sano,

Thank you for submitting your manuscript to PLOS ONE. After careful consideration, we feel that it has merit but does not fully meet PLOS ONE’s publication criteria as it currently stands. Therefore, we invite you to submit a revised version of the manuscript that addresses the points raised during the review process.

Please submit your revised manuscript by Apr 16 2026 11:59PM. If you will need more time than this to complete your revisions, please reply to this message or contact the journal office at plosone@plos.org. When you're ready to submit your revision, log on to https://www.editorialmanager.com/pone/ and select the 'Submissions Needing Revision' folder to locate your manuscript file.

Please include the following items when submitting your revised manuscript:

- A letter that responds to each point raised by the academic editor and reviewer(s). You should upload this letter as a separate file labeled 'Response to Reviewers'.
- A marked-up copy of your manuscript that highlights changes made to the original version. You should upload this as a separate file labeled 'Revised Manuscript with Track Changes'.
- An unmarked version of your revised paper without tracked changes. You should upload this as a separate file labeled 'Manuscript'.

-->If you would like to make changes to your financial disclosure, please include your updated statement in your cover letter. Guidelines for resubmitting your figure files are available below the reviewer comments at the end of this letter.

If applicable, we recommend that you deposit your laboratory protocols in protocols.io to enhance the reproducibility of your results. Protocols.io assigns your protocol its own identifier (DOI) so that it can be cited independently in the future. For instructions see: https://journals.plos.org/plosone/s/submission-guidelines#loc-laboratory-protocols. Additionally, PLOS ONE offers an option for publishing peer-reviewed Lab Protocol articles, which describe protocols hosted on protocols.io. Read more information on sharing protocols at https://plos.org/protocols?utm_medium=editorial-email&utm_source=authorletters&utm_campaign=protocols.

We look forward to receiving your revised manuscript.

Kind regards,

Maheshkumar Baladaniya

Academic Editor

PLOS One

**Journal Requirements:**

1. If the reviewer comments include a recommendation to cite specific previously published works, please review and evaluate these publications to determine whether they are relevant and should be cited. There is no requirement to cite these works unless the editor has indicated otherwise.

2. Please review your reference list to ensure that it is complete and correct. If you have cited papers that have been retracted, please include the rationale for doing so in the manuscript text, or remove these references and replace them with relevant current references. Any changes to the reference list should be mentioned in the rebuttal letter that accompanies your revised manuscript. If you need to cite a retracted article, indicate the article’s retracted status in the References list and also include a citation and full reference for the retraction notice.

**Additional Editor Comments:**

There are some concerns raised by the reviewer that needs to be addressed before accepting.

[Note: HTML markup is below. Please do not edit.]

Reviewers' comments:

Reviewer's Responses to Questions

**Comments to the Author**

1. If the authors have adequately addressed your comments raised in a previous round of review and you feel that this manuscript is now acceptable for publication, you may indicate that here to bypass the “Comments to the Author” section, enter your conflict of interest statement in the “Confidential to Editor” section, and submit your "Accept" recommendation.

Reviewer #1: All comments have been addressed

Reviewer #4: (No Response)

**********

2. Is the manuscript technically sound, and do the data support the conclusions?

The manuscript must describe a technically sound piece of scientific research with data that supports the conclusions. Experiments must have been conducted rigorously, with appropriate controls, replication, and sample sizes. The conclusions must be drawn appropriately based on the data presented.

Reviewer #1: Partly

Reviewer #4: Partly

**********

3. Has the statistical analysis been performed appropriately and rigorously?

Reviewer #1: Yes

Reviewer #4: No

**********

4. Have the authors made all data underlying the findings in their manuscript fully available?

The PLOS Data policy requires authors to make all data underlying the findings described in their manuscript fully available without restriction, with rare exception (please refer to the Data Availability Statement in the manuscript PDF file). The data should be provided as part of the manuscript or its supporting information, or deposited to a public repository. For example, in addition to summary statistics, the data points behind means, medians and variance measures should be available. If there are restrictions on publicly sharing data—e.g. participant privacy or use of data from a third party—those must be specified.

Reviewer #1: Yes

Reviewer #4: No

**********

5. Is the manuscript presented in an intelligible fashion and written in standard English?

PLOS ONE does not copyedit accepted manuscripts, so the language in submitted articles must be clear, correct, and unambiguous. Any typographical or grammatical errors should be corrected at revision, so please note any specific errors here.

Reviewer #1: Yes

Reviewer #4: Yes

**********

6. Review Comments to the Author

Please use the space provided to explain your answers to the questions above. You may also include additional comments for the author, including concerns about dual publication, research ethics, or publication ethics. (Please upload your review as an attachment if it exceeds 20,000 characters)

Reviewer #1: A significant clinical topic about bladder sensation recovery after childbirth is addressed in this updated publication, which also offers helpful service-level data. A service evaluation is a suitable study design, and the results generally corroborate the key findings. However, their reliability and generalizability are limited by their reliance on subjective patient-reported improvement and their small sample size, which should be made clearer.

Reviewer #4: The authors state in their response that they intend to redefine the self-controlled analysis as the primary analysis. While this represents a reasonable attempt to address unmeasured time-invariant confounding, several critical methodological and reporting issues must be resolved. These issues primarily concern (1) the validity of self control study design assumptions in the present clinical context, (2) inconsistency in study presentation, and (3) discordant findings across analytical frameworks.

1. Appropriateness of self control study design as the Primary Analytical Framework

Self-controlled designs, including self control study design, rely on several key assumptions. It is currently unclear whether these assumptions are satisfied in the present study.

1.1 Assumption of event-independent exposure

A fundamental assumption of self control study design is that the occurrence of the outcome does not influence the probability or timing of exposure.

In this study, pneumonia—particularly recurrent or severe pneumonia—is a major clinical indication for laryngoplasty. Consequently, pneumonia events occurring prior to surgery are likely to increase the probability of subsequent exposure (i.e., surgery). This violates the assumption of event-independent exposure and introduces event-dependent exposure (confounding by indication).

Unless explicitly addressed (for example, by excluding or separately modeling a pre-exposure high-risk window), this violation may substantially bias self control study design estimates.

1.2 Assumption of stable baseline risk

self control study design further assumes that baseline risk within individuals is either constant over time or adequately controlled through time-varying covariates.

In the present study, several strong time-varying factors—such as progression of dysphagia, increasing frailty, perioperative care, and changes in clinical surveillance—are likely to differ systematically between pre- and post-surgical periods. These factors are not fully accounted for and may confound within-person comparisons.

1.3 Conclusion regarding self control study design validity

Given the likely violation of key self control study design assumptions, particularly event-dependent exposure, the manuscript should either:

provide a detailed justification demonstrating that self control study design assumptions are reasonably met, or

clearly acknowledge that self control study design estimates may be biased and interpret them as descriptive rather than causal.

Without such clarification, using self control study design as the primary analysis is difficult to justify.

2. Implications for Baseline Characteristics (Table 1) and Study Design (Figure 1)

If the authors define the self-controlled analysis as the primary analytical framework, the presentation of baseline characteristics and study design must be revised accordingly.

2.1 Inconsistency of the current Table 1

Table 1 currently presents baseline characteristics comparing patients who did and did not undergo surgery. This structure is appropriate for a between-person cohort analysis but not for a self-controlled study, where comparisons are made within individuals.

In self control study design, between-group baseline differences are, by design, irrelevant to the primary comparison.

2.2 Recommended revision of Table 1

If self control study design is the primary analysis, Table 1 should instead describe only the population included in the self-controlled analysis, namely patients who underwent surgery.

Appropriate baseline information may include:

demographics of operated patients,

prevalence of dysphagia and other comorbidities,

frequency and severity of pneumonia prior to surgery (e.g., recurrent episodes, oxygen requirement),

timing of surgery relative to prior pneumonia events.

Similarly, Figure 1 (study flow diagram) should be revised to clearly reflect a self-controlled analytical framework rather than a between-group cohort comparison.

2.3 Overall recommendation regarding study presentation

While adopting a self-controlled design may help address unmeasured time-invariant confounding, the current study context raises serious concerns regarding the validity of self control study design assumptions, particularly because pneumonia is a major indication for surgery.

If the authors wish to proceed with self control study design as the primary analysis, they must:

explicitly justify the appropriateness of self control study design assumptions,

address event-dependent exposure and time-varying confounding, and

revise figures and baseline tables to align with a self-controlled analytic framework.

Without these revisions, self control study design results should be interpreted cautiously and may be more appropriately positioned as a secondary or sensitivity analysis.

3. Inconsistency Between the Abstract and the Declared Primary Analysis (self control study design)

The authors indicate that self control study design will be treated as the primary analysis; however, the Abstract does not describe self control study design methodology or a self-controlled design.

3.1 Issues with the current Abstract

The Abstract lacks any mention of a self-controlled or within-person study design.

Key methodological elements of self control study design (e.g., comparison of pre- and post-exposure periods within individuals) are not described.

As a result, readers may incorrectly assume that the primary analysis is a conventional cohort or between-person comparison.

3.2 Recommended revision of the Abstract

If self control study design is the primary analysis, the Abstract should explicitly state:

the use of a self-controlled or self control study design design,

that comparisons are made within individuals before and after surgery, and

the primary effect measure derived from this approach.

Failure to align the Abstract with the declared primary methodology may mislead readers and does not conform to standard reporting practices for observational studies.

4. Discordant Results Between Matched Cohort and Self-Controlled Analyses

A major concern is that the matched cohort analysis and the self-controlled analysis yield results in opposite directions:

The propensity score–matched cohort analysis suggests a higher postoperative pneumonia risk among surgically treated patients compared with matched non-surgical controls.

In contrast, the self-controlled analysis suggests a lower pneumonia incidence after surgery relative to the immediate preoperative period.

Given this discrepancy, the study cannot support a single directional conclusion regarding the effect of surgery on pneumonia risk. At a minimum, the findings indicate that postoperative pneumonia risk is highly sensitive to study design and analytical assumptions.

Accordingly, the manuscript should explicitly state that:

the direction of association differs depending on the analytical framework, and

no definitive conclusion regarding risk reduction or risk increase can be drawn from the current data.

**********

7. PLOS authors have the option to publish the peer review history of their article (what does this mean?). If published, this will include your full peer review and any attached files.

If you choose “no”, your identity will remain anonymous but your review may still be made public.

**Do you want your identity to be public for this peer review?**  For information about this choice, including consent withdrawal, please see our Privacy Policy.

Reviewer #1: No

Reviewer #4: No

**********

[NOTE: If reviewer comments were submitted as an attachment file, they will be attached to this email and accessible via the submission site. Please log into your account, locate the manuscript record, and check for the action link "View Attachments". If this link does not appear, there are no attachment files.]

To ensure your figures meet our technical requirements, please review our figure guidelines: https://journals.plos.org/plosone/s/figures

You may also use PLOS’s free figure tool, NAAS, to help you prepare publication quality figures: https://journals.plos.org/plosone/s/figures#loc-tools-for-figure-preparation.

NAAS will assess whether your figures meet our technical requirements by comparing each figure against our figure specifications.

# Author comment: Author response to Decision Letter 3

**DOI:** 10.1371/journal.pone.0352874.r006

23 Mar 2026

Thank you very much for taking the time to review our work. Your comments are appreciated and addressed below;

Note: the quotes from reviewers are in normal font, and our response is in navy blue font. The changes are highlighted in the revised manuscript.

Reviewer #1:

A significant clinical topic about bladder sensation recovery after childbirth is addressed in this updated publication, which also offers helpful service-level data. A service evaluation is a suitable study design, and the results generally corroborate the key findings. However, their reliability and generalizability are limited by their reliance on subjective patient-reported improvement and their small sample size, which should be made clearer.

Response:

We appreciate you taking the time to review our work, again. However, we are afraid that the comment seems to be about a different study (bladder sensation recovery after childbirth), which is unrelated to our manuscript on laryngoplasty and pneumonia incidence in UVFP.

Reviewer #4:

The authors state in their response that they intend to redefine the self-controlled analysis as the primary analysis. While this represents a reasonable attempt to address unmeasured time-invariant confounding, several critical methodological and reporting issues must be resolved. These issues primarily concern (1) the validity of self control study design assumptions in the present clinical context, (2) inconsistency in study presentation, and (3) discordant findings across analytical frameworks.

Response: We sincerely thank the reviewer for the thorough and methodologically insightful comments. In response to these concerns, we substantially revised the analytical framework and interpretation of our findings. Importantly, we changed the title from “Reduction in Pneumonia Incidence…” to “Association Between Laryngoplasty and Pneumonia Incidence…” to avoid causal or directional implications not fully supported by the data. We also repositioned the matched cohort analysis as the primary analysis and the self-controlled analysis as secondary, and revised the Abstract, Results, Discussion and Conclusions to explicitly acknowledge that the direction of association differed depending on the analytical framework applied.

1. Appropriateness of self control study design as the Primary Analytical Framework

Self-controlled designs, including self control study design, rely on several key assumptions. It is currently unclear whether these assumptions are satisfied in the present study.

1.1 Assumption of event-independent exposure

A fundamental assumption of self control study design is that the occurrence of the outcome does not influence the probability or timing of exposure. In this study, pneumonia—particularly recurrent or severe pneumonia—is a major clinical indication for laryngoplasty. Consequently, pneumonia events occurring prior to surgery are likely to increase the probability of subsequent exposure (i.e., surgery). This violates the assumption of event-independent exposure and introduces event-dependent exposure (confounding by indication). Unless explicitly addressed (for example, by excluding or separately modeling a pre-exposure high-risk window), this violation may substantially bias self control study design estimates.

Response: We thank the reviewer for this important comment. We agree that pneumonia occurring prior to surgery may influence the timing or indication for laryngoplasty, introducing potential event-dependent exposure. To address this concern, we conducted a sensitivity analysis excluding pneumonia events occurring within the 2 months immediately preceding surgery, along with the corresponding person-time. Although the magnitude of the reduction was attenuated (IRR increased from 0.36 to 0.55), the direction of association remained unchanged.

We have clarified this issue in the Discussion and explicitly acknowledged that violation of the event-independent exposure assumption cannot be fully excluded; therefore, the self-controlled findings should be interpreted cautiously.

Changes:

• The materials and methods section of the manuscript now contains the following sentence: ‘The self-controlled comparison of pneumonia incidence before and after laryngoplasty may be susceptible to confounding if pneumonia events occurring shortly before surgery influence the indication for laryngoplasty. Therefore, as a sensitivity analysis, we excluded pneumonia events occurring within the 2 months immediately preceding laryngoplasty, along with the corresponding person-time.’ (page 8, lines 155–159).

• The results section of the manuscript now contains the following sentence: ‘Among the remaining 163 patients, comparison of the period from the index date to 2 months before surgery with the postoperative observation period showed that the incidence of pneumonia was significantly lower after laryngoplasty than before surgery (IRR 0.55; 95% CI, 0.37–0.83; p = 0.004) (S3 Fig).’ (page 14, lines 257–259).

• The discussion section of the manuscript now contains the following sentence: ‘Regarding the self-controlled analysis, pneumonia episodes occurring shortly before laryngoplasty may influence the decision to perform surgery, resulting in event-dependent exposure and a potential overestimation of preoperative pneumonia incidence. A sensitivity analysis excluding pneumonia events within the 2 months immediately preceding surgery attenuated the magnitude of the reduction (IRR increased from 0.36 to 0.55), suggesting that the observed effect may partially reflect perioperative time-related biases.’ (page 16, lines 294–299).

1.2 Assumption of stable baseline risk

self control study design further assumes that baseline risk within individuals is either constant over time or adequately controlled through time-varying covariates. In the present study, several strong time-varying factors—such as progression of dysphagia, increasing frailty, perioperative care, and changes in clinical surveillance—are likely to differ systematically between pre- and post-surgical periods. These factors are not fully accounted for and may confound within-person comparisons.

Response: We thank the reviewer for this important point. We acknowledge that several time-varying factors, such as progression of dysphagia, frailty, perioperative care, and changes in clinical surveillance, may differ systematically between pre- and post-surgical periods. Due to the nature of the claims database, these clinical variables were not available, and changes in baseline risk over time could not be directly accounted for.

In response, we have strengthened the limitations section to explicitly acknowledge the potential influence of time-varying confounding and the need to interpret the self-controlled findings with caution.

Changes:

• The discussion section of the manuscript now contains the following sentence: ‘In addition, several strong time-varying factors—such as progression of dysphagia, increasing frailty, and changes in clinical surveillance—are likely to differ systematically between the pre- and post-surgical periods. Because these factors were not available in the present database, the observed reduction in pneumonia incidence before and after laryngoplasty should be interpreted with caution.’ (page 16, lines 299–302).

• ‘Third, self-controlled analysis may be susceptible to time-varying confounding and event-dependent exposure. Pneumonia episodes occurring shortly before surgery may have influenced the indication for laryngoplasty and patients’ clinical status may have differed systematically between the pre- and post-surgical periods.’ (page 19, lines 364–367).

1.3 Conclusion regarding self control study design validity

Given the likely violation of key self control study design assumptions, particularly event-dependent exposure, the manuscript should either: provide a detailed justification demonstrating that self control study design assumptions are reasonably met, or clearly acknowledge that self control study design estimates may be biased and interpret them as descriptive rather than causal. Without such clarification, using self control study design as the primary analysis is difficult to justify.

Response: We appreciate the reviewer’s important comment. Although we performed sensitivity analyses to mitigate potential event-dependent exposure, we agree that violation of key assumptions of the self-controlled design cannot be fully excluded. In response, we have repositioned the matched cohort analysis as the primary analysis and treated the self-controlled analysis as secondary. We have also revised the manuscript to interpret the self-controlled findings as descriptive rather than causal and to explicitly acknowledge their susceptibility to bias.

Changes:

• The introduction section of the manuscript now contains the following sentence: ‘Understanding the clinical outcomes of laryngoplasty may help determine the indications for surgical treatment in the management of UVFP. In this study, we conducted a cross-institutional analysis using a Japanese insurance claims database to evaluate the association between laryngoplasty and pneumonia incidence in patients with UVFP.’ (page 4, lines 71–74)

• The material and methods section of the manuscript now contains the following sentence: ‘As the primary analysis, we compared the cumulative incidence of pneumonia between the treatment group and the non-treatment group, with follow-up starting from a predefined index date, defined as the onset of UVFP.’ (page 6, lines 117–119) ‘As the secondary analysis, we conducted a self-controlled analysis within the treatment group to compare the incidence of pneumonia before and after laryngoplasty.’ (page 6, lines 120–122)

• The discussion section of the manuscript now contains the following sentence: ‘These limitations may have biased within-person comparisons. As described previously, the observed reduction in pneumonia incidence after surgery should therefore be interpreted with caution.’ (page 19, lines 367-369).

2. Implications for Baseline Characteristics (Table 1) and Study Design (Figure 1)

If the authors define the self-controlled analysis as the primary analytical framework, the presentation of baseline characteristics and study design must be revised accordingly.

2.1 Inconsistency of the current Table 1

Table 1 currently presents baseline characteristics comparing patients who did and did not undergo surgery. This structure is appropriate for a between-person cohort analysis but not for a self-controlled study, where comparisons are made within individuals. In self control study design, between-group baseline differences are, by design, irrelevant to the primary comparison.

Response: We thank the reviewer for this comment. As noted above, we have revised the manuscript to designate the matched cohort analysis as the primary analytical framework. Accordingly, the presentation of baseline characteristics comparing the treatment and non-treatment groups in Table 1 is consistent with the primary between-group design. We have clarified this structure throughout the manuscript to ensure alignment between the analytical framework and the presentation of baseline characteristics.

2.2 Recommended revision of Table 1

If self control study design is the primary analysis, Table 1 should instead describe only the population included in the self-controlled analysis, namely patients who underwent surgery. Appropriate baseline information may include: demographics of operated patients, prevalence of dysphagia and other comorbidities, frequency and severity of pneumonia prior to surgery (e.g., recurrent episodes, oxygen requirement), timing of surgery relative to prior pneumonia events. Similarly, Figure 1 (study flow diagram) should be revised to clearly reflect a self-controlled analytical framework rather than a between-group cohort comparison.

Response: We appreciate the reviewer’s suggestion. As described above, we have revised the analytical framework to designate the matched cohort analysis as the primary analysis. Therefore, Table 1 appropriately presents baseline characteristics comparing the treatment and non-treatment groups, and Figure 1 reflects the between-group cohort design. We have revised the manuscript to clearly describe this structure to avoid ambiguity regarding the primary analytical framework.

2.3 Overall recommendation regarding study presentation

While adopting a self-controlled design may help address unmeasured time-invariant confounding, the current study context raises serious concerns regarding the validity of self control study design assumptions, particularly because pneumonia is a major indication for surgery. If the authors wish to proceed with self control study design as the primary analysis, they must: explicitly justify the appropriateness of self control study design assumptions, address event-dependent exposure and time-varying confounding, and revise figures and baseline tables to align with a self-controlled analytic framework. Without these revisions, self control study design results should be interpreted cautiously and may be more appropriately positioned as a secondary or sensitivity analysis.

Response: We thank the reviewer for this comprehensive recommendation. In response, we have repositioned the matched cohort analysis as the primary analysis and treated the self-controlled analysis as secondary. We have also revised the Abstract, Methods and Discussion to explicitly acknowledge the susceptibility of the self-controlled design to event-dependent exposure and time-varying confounding, and to interpret these findings cautiously. We believe these revisions align the study presentation with the reviewer’s recommendations and clarify the analytical hierarchy of the manuscript.

Changes:

• The abstract section of the manuscript now contains the following sentence: ‘The primary analysis compared the cumulative incidence of pneumonia between treatment and non-treatment groups. The secondary analysis employed a self-controlled design to compare pneumonia incidence before and after laryngoplasty within treated patients.’ (page 2, lines 37–40)

• The material and methods section of the manuscript now contains the following sentence: ‘As the primary analysis, we compared the cumulative incidence of pneumonia between the treatment group and the non-treatment group, with follow-up starting from a predefined index date, defined as the onset of UVFP.’ (page 6, lines 117–119) ‘As the secondary analysis, we conducted a self-controlled analysis within the treatment group to compare the incidence of pneumonia before and after laryngoplasty.’ (page 6, lines 120–122)

• The discussion section of the manuscript now contains the following sentence: ‘Thus, our study adds to the existing literature by examining the relationship between laryngoplasty and pneumonia incidence using multiple analytical approaches within a large insurance database.’ (page 15, lines 286-288)

• The discussion section of the manuscript now contains the following sentence: ‘Regarding the self-controlled analysis, pneumonia episodes occurring shortly before laryngoplasty may influence the decision to perform surgery, resulting in event-dependent exposure and a potential overestimation of preoperative pneumonia incidence. A sensitivity analysis excluding pneumonia events within the 2 months immediately preceding surgery attenuated the magnitude of the reduction (IRR increased from 0.36 to 0.55), suggesting that the observed effect may partially reflect perioperative time-related biases. In addition, several strong time-varying factors—such as progression of dysphagia, increasing frailty, and changes in clinical surveillance—are likely to differ systematically between the pre- and post-surgical periods. Because these factors were not available in the present database, the observed reduction in pneumonia incidence before and after laryngoplasty should be interpreted with caution.’ (page 16, lines 294-302)

3. Inconsistency Between the Abstract and the Declared Primary Analysis (self control study design)

3.1 Issues with the current Abstract

The Abstract lacks any mention of a self-controlled or within-person study design. Key methodological eleme

Attachment Submitted filename: Author responce to reviewers 20260321.docx

# Peer review: Decision Letter 3

**DOI:** 10.1371/journal.pone.0352874.r007

**Authors:** Maheshkumar Baladaniya, Maheshkumar Baladaniya, Maheshkumar Baladaniya, Patrick Goymer

17 Jun 2026

Association Between Laryngoplasty and Pneumonia Incidence in Patients With Unilateral Vocal Fold Paralysis: A Japanese Insurance Claims Database Study

PONE-D-25-45734R3

Dear Dr. Sano,

We’re pleased to inform you that your manuscript has been judged scientifically suitable for publication and will be formally accepted for publication once it meets all outstanding technical requirements.

Within one week, you’ll receive an e-mail detailing the required amendments. When these have been addressed, you’ll receive a formal acceptance letter and your manuscript will be scheduled for publication.

An invoice will be generated when your article is formally accepted. Please note, if your institution has a publishing partnership with PLOS and your article meets the relevant criteria, all or part of your publication costs will be covered. Please make sure your user information is up-to-date by logging into Editorial Manager at Editorial Manager® and clicking the ‘Update My Information' link at the top of the page. For questions related to billing, please contact billing support.

If your institution or institutions have a press office, please notify them about your upcoming paper to help maximize its impact. If they’ll be preparing press materials, please inform our press team as soon as possible -- no later than 48 hours after receiving the formal acceptance. Your manuscript will remain under strict press embargo until 2 pm Eastern Time on the date of publication. For more information, please contact onepress@plos.org.

Kind regards,

Patrick Goymer

Staff Editor

PLOS One

Additional Editor Comments (optional):

Reviewers' comments:

Reviewer's Responses to Questions

**Comments to the Author**

1. If the authors have adequately addressed your comments raised in a previous round of review and you feel that this manuscript is now acceptable for publication, you may indicate that here to bypass the “Comments to the Author” section, enter your conflict of interest statement in the “Confidential to Editor” section, and submit your "Accept" recommendation.

Reviewer #4: All comments have been addressed

**********

2. Is the manuscript technically sound, and do the data support the conclusions?

The manuscript must describe a technically sound piece of scientific research with data that supports the conclusions. Experiments must have been conducted rigorously, with appropriate controls, replication, and sample sizes. The conclusions must be drawn appropriately based on the data presented.

Reviewer #4: Yes

**********

3. Has the statistical analysis been performed appropriately and rigorously?

Reviewer #4: Yes

**********

4. Have the authors made all data underlying the findings in their manuscript fully available?

The PLOS Data policy requires authors to make all data underlying the findings described in their manuscript fully available without restriction, with rare exception (please refer to the Data Availability Statement in the manuscript PDF file). The data should be provided as part of the manuscript or its supporting information, or deposited to a public repository. For example, in addition to summary statistics, the data points behind means, medians and variance measures should be available. If there are restrictions on publicly sharing data—e.g. participant privacy or use of data from a third party—those must be specified.

Reviewer #4: Yes

**********

5. Is the manuscript presented in an intelligible fashion and written in standard English?

PLOS ONE does not copyedit accepted manuscripts, so the language in submitted articles must be clear, correct, and unambiguous. Any typographical or grammatical errors should be corrected at revision, so please note any specific errors here.

Reviewer #4: Yes

**********

6. Review Comments to the Author

Please use the space provided to explain your answers to the questions above. You may also include additional comments for the author, including concerns about dual publication, research ethics, or publication ethics. (Please upload your review as an attachment if it exceeds 20,000 characters)

Reviewer #4: (No Response)

**********

7. PLOS authors have the option to publish the peer review history of their article (what does this mean?). If published, this will include your full peer review and any attached files.

If you choose “no”, your identity will remain anonymous but your review may still be made public.

**Do you want your identity to be public for this peer review?**  For information about this choice, including consent withdrawal, please see our Privacy Policy.

Reviewer #4: No

**********

# Editor report: Acceptance letter

**DOI:** 10.1371/journal.pone.0352874.r008

**Authors:** Maheshkumar Baladaniya, Maheshkumar Baladaniya, Maheshkumar Baladaniya, Patrick Goymer

PONE-D-25-45734R3

PLOS One

Dear Dr. Sano,

I'm pleased to inform you that your manuscript has been deemed suitable for publication in PLOS One. Congratulations! Your manuscript is now being handed over to our production team.

At this stage, our production department will prepare your paper for publication. This includes ensuring the following:

* All references, tables, and figures are properly cited

* All relevant supporting information is included in the manuscript submission,

* There are no issues that prevent the paper from being properly typeset

You will receive further instructions from the production team, including instructions on how to review your proof when it is ready. Please keep in mind that we are working through a large volume of accepted articles, so please give us a few days to review your paper and let you know the next and final steps.

Lastly, if your institution or institutions have a press office, please let them know about your upcoming paper now to help maximize its impact. If they'll be preparing press materials, please inform our press team within the next 48 hours. Your manuscript will remain under strict press embargo until 2 pm Eastern Time on the date of publication. For more information, please contact onepress@plos.org.

You will receive an invoice from PLOS for your publication fee after your manuscript has reached the completed accept phase. If you receive an email requesting payment before acceptance or for any other service, this may be a phishing scheme. Learn how to identify phishing emails and protect your accounts at https://explore.plos.org/phishing.

If we can help with anything else, please email us at customercare@plos.org.

Thank you for submitting your work to PLOS ONE and supporting open access.

Kind regards,

PLOS ONE Editorial Office Staff

on behalf of

Dr Patrick Goymer

Staff Editor

PLOS One
